# The Crosstalk with CXCL10‐Rich Tumor‐Associated Mast Cells Fuels Pancreatic Cancer Progression and Immune Escape

**DOI:** 10.1002/advs.202417724

**Published:** 2025-02-18

**Authors:** Hanlin Yin, Qiangda Chen, Shanshan Gao, Sami Shoucair, Yuqi Xie, Joseph R. Habib, Taochen He, Wei Gan, Juan Wang, Lei Zhang, Huaxiang Xu, Chenye Shi, Junyi He, Wenquan Wang, Yun Jin, Michael G Goggins, Liang Liu, Wenhui Lou, Wenchuan Wu, Jun Yu, Ning Pu

**Affiliations:** ^1^ Department of Pancreatic Surgery Zhongshan Hospital Fudan University Shanghai 200032 China; ^2^ Cancer Center Zhongshan Hospital Fudan University Shanghai 200032 China; ^3^ Department of Radiology Zhongshan Hospital Fudan University Shanghai 200032 China; ^4^ Departments of Medicine, Oncology and Surgery Johns Hopkins University School of Medicine Baltimore MD 21287 USA; ^5^ Department of Surgery New York University School of Medicine and NYU‐Langone Medical Center New York NY 10016 USA; ^6^ HANGZHOU CHEXMED TECHNOLOGY CO., LTD Hangzhou 310000 China; ^7^ Department of Hepatobiliary and Pancreatic Surgery The First People's Hospital of Yunnan Province The Affiliated Hospital of Kunming University of Science and Technology Kunming 650500 China; ^8^ Departments of Medicine and Pathology The Sol Goldman Pancreatic Cancer Research Center Johns Hopkins University School of Medicine Baltimore MD 21287 USA; ^9^ Pancreas Center Tianjin Medical University Cancer Institute & Hospital Tianjin Medical University Tianjin 300060 China

**Keywords:** CXCL10, immune escape, pancreatic ductal adenocarcinoma, sodium cromoglycate, tumor‐associated mast cell

## Abstract

Pancreatic ductal adenocarcinoma (PDAC) is a devastating disease, necessitating approaches to improve prognosis. As the mediator of allergic process, mast cells have been found in various cancers and are associated with survival. However, the biological behaviors of tumor‐associated mast cells (TAMCs) remain unclear. Herein, an excessive infiltration of TAMCs in PDAC is demonstrated, which apparently associated with poor survival in PDAC patients. PDAC cells are found to recruit CXCR2^+^ MCs into TME, and then inhibited MCs ferroptosis, and maintained their proliferation. Concomitantly, the tumor‐derived exosome miR‐188‐5p activated the PTEN/AKT/GSK3β signaling, further stabilized transcriptional factor ERG by inhibiting its ubiquitin degradation, and finally enhanced the transcription of *cxcl10* within TAMCs. In reverse, TAMCs‐derived CXCL10 reversely promoted tumor epithelial‐mesenchymal transition and induced immunosuppressive tumor microenvironment by recruiting CXCR3^+^ Tregs. Sodium cromoglycate (SCG) is a membrane stabilizer for MCs and confirmed as an effective and widely used agent to block TAMCs‐derived CXCL10 and further sensitize the therapeutic efficacy of anti‐PD‐1 antibody plus gemcitabine for PDAC. These findings illuminate a critical and innovative crosstalk between TAMCs and PDAC cells that promote PDAC progression, and SCG sensitizes PDAC to the current immuno‐chemotherapy, which reveals its potential to be a valuable adjuvant for PDAC patients.

## Graphical abstract

Pancreatic cancer promotes the accumulation of mast cells in the tumor microenvironment. Tumor‐derived exosomes deliver miR‐188‐5p to tumor‐associated mast cells (TAMCs), enhancing TAMCs‐derived CXCL10 secretion by strengthening ERG transcription. TAMCs‐derived CXCL10 promotes tumor migration, stemness, and immunosuppressive environment. Sodium cromoglycate disrupts TAMCs‐tumor crosstalk by inhibiting the secretion of CXCL10 from TAMCs, thereby sensitizing the therapeutic effects of immuno‐chemotherapy.

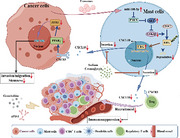

## 1 Introduction

Pancreatic ductal adenocarcinoma (PDAC) is a highly malignant type of cancer characterized by its resistance to chemotherapy and radiation therapy. Currently, it stands as the estimated third leading cause of cancer‐related death in the United States, with only a 13% 5‐year survival rate.^[^ ^1^ ^]^ Despite advancements in PDAC treatment, long‐term survival rates have shown limited improvements over the past few decades. Therefore, it is imperative to better understand the characteristics of PDAC to explore novel and effective strategies to improve the current therapeutic efficacy.

There is a growing belief that the intricate interplay between tumor cells and the surrounding microenvironment significantly fuels tumor progression and affects patient outcomes.^[^ ^2^ ^]^ PDAC is notable for its distinct histological characteristics, which encompass intense stroma desmoplasia and an abundance of fibroblasts, immune cells, and aberrant vascular structures. Stroma and immune cells actually augment the malignant attributes of tumor cells.^[^ ^3^ ^]^ Among them, myeloid lineage cells make up the largest immune cell population in PDAC tumor, which have a powerful impact on PDAC patient outcomes. For instance, myeloid‐derived suppressor cells (MDSCs) or tumor‐associated macrophages (TAMs) can establish a tolerant tumor microenvironment (TME) by manipulating T cells phenotype and disrupting the immuno‐oncology cycle.^[^ ^4^ ^]^

Mast cells (MCs) constitute vital components of myeloid cells, and participate in allergy by secreting histamine and protease. Additionally, MCs modulate tissue repair, angiogenesis, and immune responses by interacting with other cells.^[^ ^5^ ^]^ Recently, MCs have gained attention for their role in malignancies. Tumor‐infiltrating MCs actively participate in tumor progression, and are associated with poor prognosis in various cancers, such as cholangiocarcinoma,^[^ ^6^ ^]^ breast cancer,^[^ ^7^ ^]^ and gastric cancer.^[^ ^8^ ^]^ Although the relationship between MCs and tumorigenesis remains controversial in PDAC mouse model,^[^ ^9^ ^]^ it is undisputed that high tumor MCs infiltration was correlated with poor survival, demonstrating the pro‐tumoral capacity of MCs in progressed tumor.^[^ ^10^ ^]^ However, the potential mechanism by which MCs promote tumor progression in PDAC remains unclear and requires further study.

Transcriptional analysis of MCs’ originating from various tissues has revealed their transcriptional diversity^[^ ^11^ ^]^ Moreover, within the same tissue, the heterogeneous distribution of cytokines can give rise to distinct subpopulations, also shown the heterogeneity of mast cells.^[^ ^12^ ^]^ Thus, the unique transcriptional characteristics of tumor‐educated MCs, known as tumor‐associated mast cells (TAMCs), needs to be clear in PDAC. In this study, we have demonstrated that PDAC patients with a high density of TAMCs in the TME had poor outcomes. PDAC cells recruited CXCR2^+^MCs, and orchestrated their biological behavior, resulting in the accumulation in TME. Furthermore, tumor‐derived exosome miR‐188‐5p promoted the transcription of CXCL10 in TAMCs via the PTEN/AKT/GSK3β/ERG axis. Reversely, CXCL10 produced by TAMCs directly enhanced cancer cell migration and stemness through PPARγ signaling, and promoted immune escape via recruiting immunosuppressive Tregs. Finally, sodium cromoglycate (SCG), a membrane stabilizer, suppressed PDAC dissemination, and enhanced sensitivity to the combination therapy of αPD‐1 antibody and gemcitabine. Our findings demonstrate the intricate crosstalk between TAMCs and PDAC cells, and propose SCG as a potential modifier for the current immuno‐chemotherapy in PDAC.

## 2 Results

### 2.1 Mast Cell Density is Increased in PDAC Tissues and Correlates with Poor Prognosis

**Figure 1:**
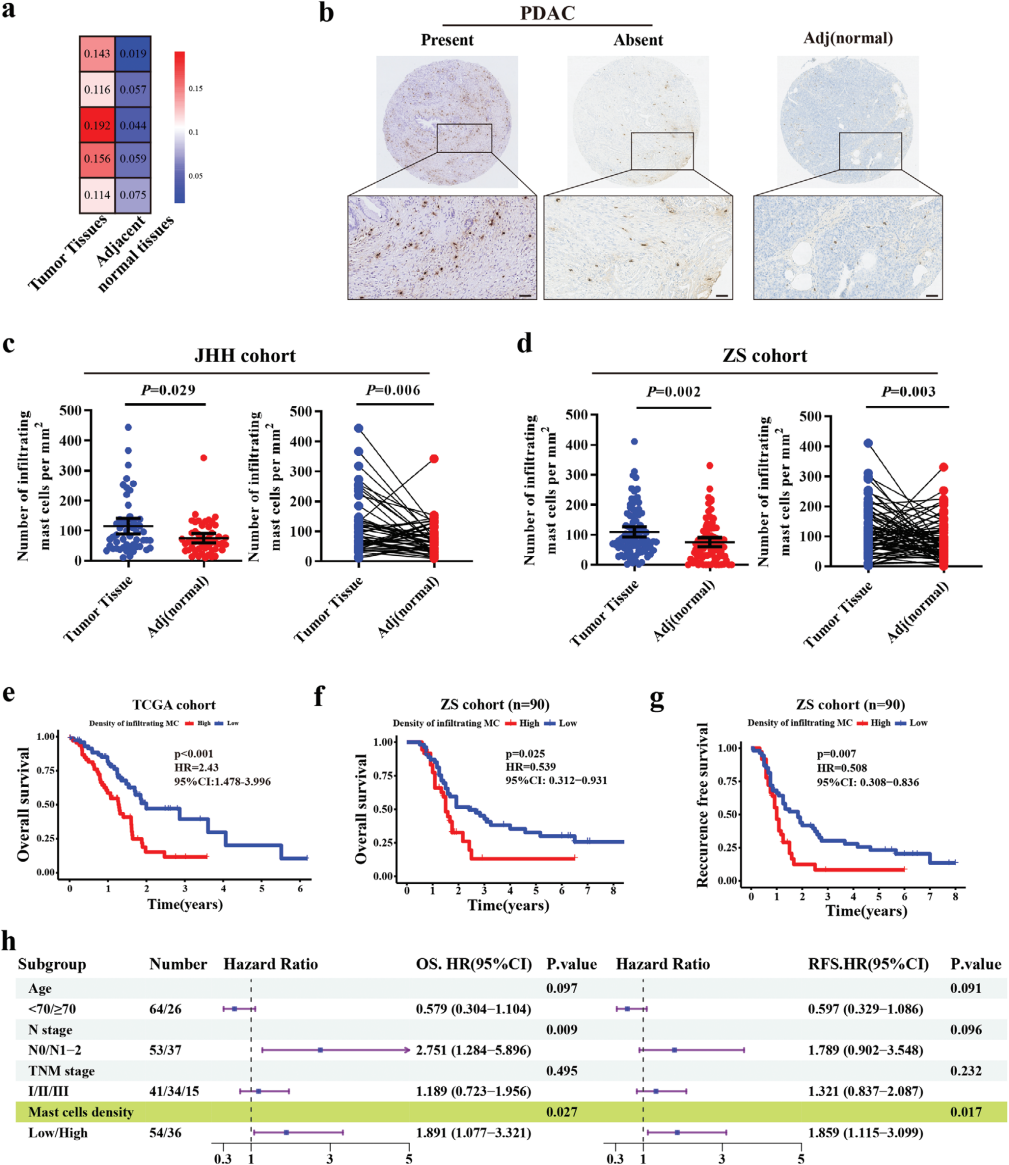
Mast cells excessively accumulate in PDAC tissues and correlate with poor prognosis. a) Mast cell infiltration analysis from transcriptome data of five PDAC tissues and paired adjacent normal tissues via CIBERSORT analysis. b) Representative images of TPSAB1 staining in PDAC tissues and adjacent normal tissues. Scale bar: 200µm. c) and d) Quantification and comparison of mast cells in tumor tissues and adjacent normal tissues from JHH (Johns Hopkins Hospital) cohort (c) and ZS (Zhongshan Hospital) cohort (d) by two‐sample t test and paired t test analysis, respectively. e) The Kaplan‐Meier plot of overall survival in PDAC stratified by mast cell density using CIBERSORT algorithm from TCGA database. f) and g) Kaplan‐Meier plot of overall survival (f) and recurrence free survival (g) in PDAC from the ZS cohort stratified by mast cells density. h) The visualization of independent prognostic factor for overall survival and recurrence free survival in the ZS cohort via multivariate Cox regression analysis.

To investigate the TAMCs infiltration in PDAC, we reanalyzed previous RNA‐seq data from five PDAC tissues and their corresponding adjacent normal tissues using CIBERSORT analysis.^[^ ^13^ ^]^ The analysis revealed a significant accumulation of TAMCs in PDAC tissues compared to their adjacent normal tissues (**Figure** **1**a). Toluidine blue, c‐kit, and TBSAB1 were stained in PDAC for mast cell identification. Among them, TPSAB1 exhibited the most distinct and identifiable staining (Figure S1, Supporting Information). The distribution of TAMCs was further validated in tissue microarrays (TMAs) from two separate tertiary medical centers. The results revealed that TAMCs were primarily distributed in the tumor interstitium (Figure 1b), and they were significantly enriched in tumor tissues compared to their adjacent normal tissues in both ZS and JHH cohorts (Figure 1c,d). PDAC patients with higher TAMCs infiltration were also found to have significantly shorter overall survival (OS) than those with lower infiltration in TCGA cohort (Figure 1e).

The clinical relevance and prognosis of TAMCs were further validated in the ZS cohort. The entire cohort was divided into the TAMC^hi^ and TAMC^low^ group according to the calculated optimal cut‐off value (100.5 cells). Under the correlation analysis, only tumor differentiation was significantly associated with TAMCs density in PDAC (P = 0.020), while no significant relationship was found with others (Table S1, Supporting Information). The Kaplan‐Meier analysis revealed that the median OS (18.0 versus 29.0 months, P = 0.025, Figure 1f) and recurrence‐free survival (RFS, 12.0 versus 22.0 months, P = 0.007, Figure 1g) in the TAMC^hi^ group were significantly shorter than those in the TAMC^low^ group. After adjusted analysis, TAMCs density in PDAC was finally eventually identified as the only significant independent risk indicator for both OS (P = 0.027, HR = 1.891, 95%CI: 1.077‐3.321) and RFS (P = 0.017, HR = 1.859, 95%CI: 1.115‐3.099) (Figure 1h; Table S2, Supporting Information). Collectively, our results demonstrated that higher density of TAMCs within TME was correlated with poor outcome in PDAC patients.

### 2.2 Pancreatic Cancer Cells Promote Migration and Sustain Proliferation of Mast Cells

**Figure 2:**
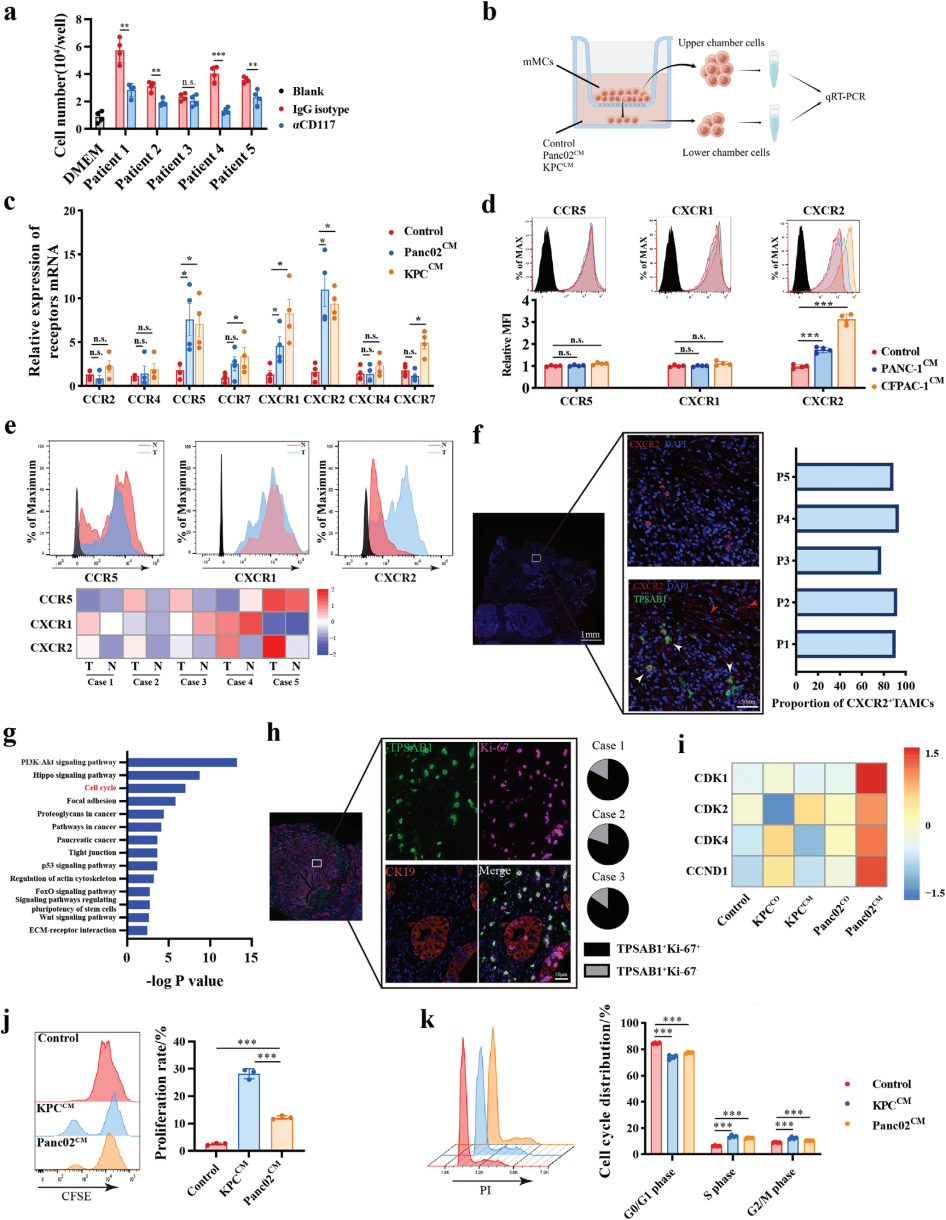
PDAC recruits mast cell infiltration and promotes its proliferation. a) Mast cell migration assay comparing the recruitment ability of tumor‐conditioned medium. Surgical resected PDAC tissues (n = 5) were digested into single cells and resuspended by DMEM. After culturing for 24h, supernatant was harvested as tumor‐conditioned medium. Anti‐CD117 was used at 10µg mL^−1^ to block SCF/CD117 signaling. b) Schematic representation of mast cell migration assay and qRT‐PCR analysis. Mast cells were inoculated into the upper chamber for 12 h and collected from both upper and lower chambers. c) Relative expression of chemokine receptors in migrated mMCs via qRT‐PCR analysis. The expression of chemokine receptors was calculated by ΔΔCT^upper^/ΔΔCT^upper^ and normalized to control groups. n = 4 per group. d) Representative histogram and statistical analysis of CCR5, CXCR1, and CXCR2 expression on migrated hMCs via flow cytometry. n = 4 per group. e) Representative histogram images and normalized heatmap of chemokine receptor expression in tumor‐associated mast cells in PDAC (n = 5). Mast cells were gated on CD45^+^, CD117^+^, and FcεRI^+^. f) Representative immunofluorescence images of CXCR2 and TPSAB1 co‐staining in PDAC tissues and quantification for the ratio of CXCR2^+^TAMCs to total TAMCs. TPSAB1, green; CXCR2, red; DAPI, blue. Red arrow: CXCR2^+^TPSAB1^−^ cells; White arrow: CXCR2^+^TPSAB1^+^ TAMCs; Green arrow: CXCR2^−^TPSAB1^+^ MCs; Scale bars: 100µm. g) KEGG pathways were enriched based on DGEs from mast cells co‐cultured with tumor cells or not. h) Proliferated tumor‐associated mast cells were defined as Ki‐67^+^ TPSAB1^+^ double positive cells by immunofluorescence staining. TPSAB1, green; Ki‐67, pink; cytokeratin 19, orange; DAPI, blue. Scale bars: 50µm. i) Heatmap of proliferation‐related gene expressions from transcriptional RNA sequencing for mast cell following indicated treatments. j) Representative histogram of CFSE staining and the ratios of proliferative cells to total cells from indicated groups. n = 3 per group. mMCs were labeled by CFSE and stimulated by tumor‐conditioned medium or DMEM as control for 48 hours. k) Representative histogram of cell cycle staining and the statistical analysis of the percentage in each cell cycle phase from indicated groups. mMCs were stimulated by tumor‐conditioned medium or DMEM as control for 48 hours, and then stained by PI for cell cycle analysis. n = 4 per group. *P < 0.05, **P < 0.01, and ***P < 0.001, Data were displayed as mean ± SD.

Stem cell factor (SCF) plays a crucial role in MC proliferation, survival, and chemotaxis.^[^ ^14^ ^]^ We found that PDAC cell lines constitutively expressed SCF (Figure S2a, Supporting Information). Moreover, SCF expression in tumor positively correlated with TAMCs infiltration, as determined by xCell and CIBERSORT (Figure S2b, Supporting Information), which suggested that SCF might participated in TAMCs recruitments. To ascertain this hypothesis, human peripheral mast cells (hMCs) were cultured in vitro (Figure S2c, Supporting Information) and confirmed as c‐kit^+^FcεR1^+^ cells by flow cytometry (Figure S2d, Supporting Information). Blocking SCF/CD117 axis on hMCs by CD117 antibody could partially reverse the recruitment ability induced by tumor‐conditioned medium (**Figure** **2**a), which is consistent with previous studies.^[^ ^15^ ^]^ This indicates that SCF is partially involved in the recruitment of MCs in PDAC.

It has been reported that CXCR family also participates in MCs migration in gastric cancer.^[^ ^16^ ^]^ To further explore the potential mechanism, murine bone marrow‐derived mast cells (mMCs) were expanded in vitro (Figure S3a, b, Supporting Information), and identified by immunostaining (Figure S3c, Supporting Information) and flow cytometry (Figure S3d, Supporting Information). Similarly, a significant chemotaxis of mMCs was observed when co‐cultured with KPC and Panc02 cell lines (Figure S3e, Supporting Information).

To identify the specific chemokine receptors involved in mast cell migration, a migration assay was performed as previously reported^[^ ^16^ ^]^ (Figure 2b). The results showed that *ccr5*, *cxcr1*, and *cxcr2* were highly expressed in the low‐chamber mMCs in both murine PDAC cell lines, suggesting their potential role in the chemotaxis process (Figure 2c), but only CXCR2^+^hMCs were recruited by human PDAC cell lines (Figure 2d). Similarly, TAMCs had higher CXCR2 expression when compared to MCs in adjacent normal pancreas (Figure 2e). CXCR2 is an essential chemotactic receptor in myeloid cells. The dual immunofluorescence analysis revealed that majority of TAMCs strongly expressed CXCR2 in tumor lesion (Figure 2f). CXCR2 receptor blockade significantly reversed the chemotaxis effect of PDAC conditioned medium (Figure S3f, Supporting Information). Six potential chemokines that could interact with CXCR2 were upregulated in tumor tissues in most public databases, particularly CXCL5 and CXCL8 (Figure S3g, h, Supporting Information). Thus, these findings collectively suggest that pancreatic cancer could recruit CXCR2^+^MCs into TME and exert pro‐tumor biological behavior.

The RNA‐seq revealed a significant enrichment of cell cycle pathway MCs co‐cultured with PDAC cells, indicating that tumor cells may influence the proliferation of MCs (Figure 2g). Furthermore, the dual immunofluorescence analysis showed that a majority of TAMCs exhibited abundant expression of Ki‐67, suggesting their increased proliferative ability in the TME (Figure 2h). Additionally, the cell cycle related genes, including CDK family and CCND1, were significantly upregulated in MCs after tumor education (Figure 2i). Then, the increased proliferative ability of MCs stimulated by tumor‐conditioned medium also verified by flow cytometry in vitro (Figure 2j,k). Traditionally, MCs can be activated by IgE and play a traditional role in allergic disorder by secreting inflammatory mediators, including arachidonic acid metabolic products, histamine, and inflammatory cytokines.^[^ ^17^ ^]^ However, we found that TAMCs downregulated the expression of the IgE receptor, *Fcer2a*. Additionally, the expression of phospholipase A2 initiation enzyme (*pla2g5*), leukotrienes‐related enzymes (*ltc4s* and *lta4h*), or prostaglandin‐related enzymes (*ptgs2* and *ptgds*) were also decreased in TAMCs. Cytokines from the interleukin family, which are important in IgE‐related inflammation, were also decreased in TAMCs (Figure S3i, Supporting Information). Degranulation assay also demonstrated that TAMCs had lower histamine release ability (Figure S3j, Supporting Information). These results indicated that TAMCs had lost their traditional allergic response characteristics and exhibited unique biological behaviors instead.

### 2.3 Pancreatic Cancer Cell‐Derived Exosomes Restrain Mast Cell Ferroptosis through PI3K/AKT Signaling

**Figure 3:**
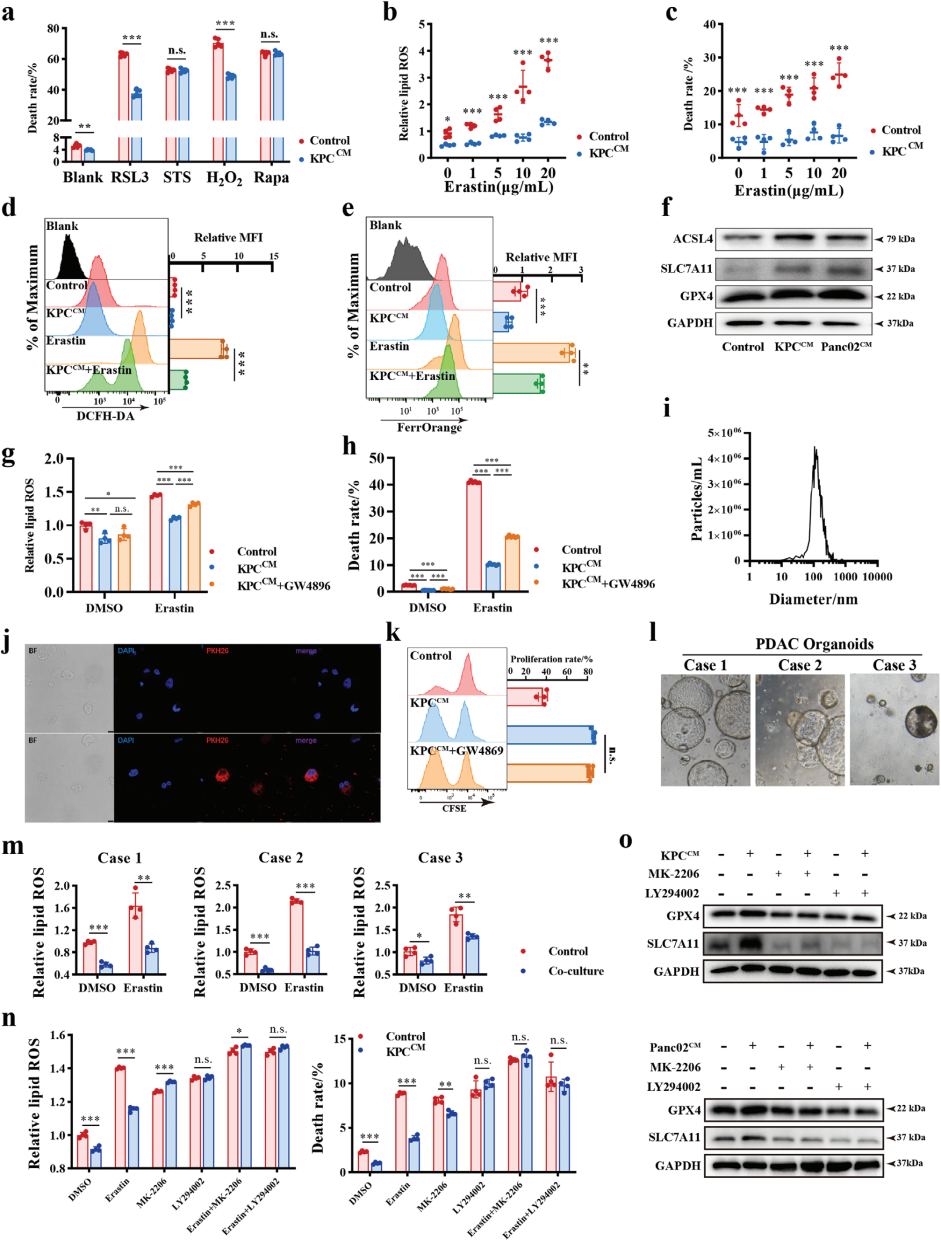
Tumor‐conditioned medium inhibits mast cell ferroptosis via AKT pathway activation. a) The death rate of indicated cells following treatment with different cell death inducers. RSL3: ferroptosis activator; Staurosporine(STS): apoptosis activator; hydrogen peroxide(H_2_O_2_): ROS stress inducer; Rapamycine(Rapa): autophagy inducer). Cells were pre‐treated by KPC‐conditioned medium or DMEM as control for 12 h, then stimulated by death inducers for 24 h, and stained by PI for evaluation of cell death. n = 5 per group. b) and c) The lipid ROS levels (b) and death rates (c) were compared between mast cells treated by KPC‐conditioned medium or not for 24 h. d) and e) Flow cytometry analysis for ROS levels (d) and Fe^2+^ levels (e) in mast cells following indicated treatments. n = 4 per group. f) Western blot analysis for ACSL4, SLC7A11, and GPX4 expression in mast cells after indicated treatment for 24 h. g) and h) Flow cytometry analysis for lipid ROS levels (n = 4). g) and cell death rates (n = 6). h) in mast cells after conditioned medium with or without exosomes. i) Histogram showing the diameter distribution of KPC‐derived exosomes. j) KPC‐derived exosomes were pre‐stained by PKH26 (membrane dye for cell‐tracing assay) and used for exosome intake assay by co‐culturing with mast cells. k) Flow cytometry analysis for mast cell proliferation by CFSE after indicated treatment for 48 hours. n = 4 per group. l) Representative image of three PDAC patient‐derived organoids. m) Mast cells co‐cultured with three patient‐derived organoids and then treated with Erastin to detect lipid ROS levels. n = 4 per group. n) The effect of AKT pathway inhibitors including MK‐2206 (AKT phosphorylation inhibitor, 3µM) and LY294002 (PI3K inhibitor, 10µM) on lipid ROS (left panel) and cell death rate (right panel) of mast cells after the treatment of Erastin and KPC‐conditioned medium. n = 4 per group. o) The expression of GPX4 and SLC7A11 in mast cells after they were pre‐treated with AKT inhibitors and stimulated by KPC‐conditioned medium (upper) or Panc02‐conditioned medium (lower). n.s. *P* > 0.05, *P < 0.05, **P < 0.01, and ***P < 0.001, Data were displayed as mean ± SD.

To confirm whether pancreatic cancer cells could inhibit the death of MCs, we applied different cell death inducers as previously reported.^[^ ^18^ ^]^ Our results showed that PDAC cell‐conditioned medium effectively rescued cell death induced by H_2_O_2_^[^ ^19^ ^]^ and RSL3,^[^ ^20^ ^]^ suggesting that tumor‐conditioned medium might protect MCs from ferroptosis (**Figure** **3**a). Subsequently, KPC‐conditioned medium was shown to reduce Erasin‐induced lipid ROS accumulation (Figure 3b) and cell death (Figure 3c) in MCs, and this effect was similarly observed in Panc02‐conditioned medium (Figure S4a, Supporting Information). In addition to lipid ROS accumulation and elevated cell death, intracellular ROS and Fe^2+^ are also indicators of ferroptosis. Pretreatment of MCs with KPC supernatant significantly rescued the intracellular ROS (Figure 3d) and Fe^2+^ ion (Figure 3e; Figure S4b, Supporting Information) induced by Erastin in mMCs.

Ferroptosis is governed by the process of membrane lipid peroxidation and inner defenses. We analyzed the expression of three core proteins as the representation for lipid hydroperoxides generation and elimination process in ferroptosis. Tumor‐conditioned medium significantly upregulated cystine/glutamate transporter (solute carrier family 7 member 11, SLC7A11) and the key enzyme for lipid hydroperoxide elimination (glutathione peroxidase 4, GPX4) expression in mMCs, both of which negatively regulate ferroptosis by importing L‐cystine and catalyzing the degradation of lipid peroxides (Figure 3f). However, Acyl‐CoA synthetase long chain family member 4 (ACSL4), which participates in polyunsaturated fatty acid‐containing phospholipids (PUFA‐PLs) synthesis, was also found to be aberrantly increased after tumor conditioned medium treatment.

Given that MCs are primarily located in the stroma and distant from PDAC cells, we hypothesized that tumor cells may educate TAMCs in non‐contact dependent way. To determine which pathway mainly mediated this process, N‐Smase inhibitor GW4869 was utilized to block exosome secretion. We observed that the protective effect of tumor‐conditioned medium could be partially blocked by GW4869, suggesting the involvement of exosomes derived from KPC (Figure 3g,h) and Panc02 (Figure S4c, d, Supporting Information) in this process. The KPC‐derived exosomes were separated by ultracentrifugation and further identified (Figure 3i; Figure S4e, f, Supporting Information). PKH26 labeled tracking assay showed the uptake of KPC‐derived exosomes by MCs (Figure 3j). Exosome depletion in tumor conditioned medium did not impair mMCs proliferation, revealing that PDAC cells promoting MCs proliferation are exosome‐independent (Figure 3k; Figure S4g, Supporting Information). Moreover, three PDAC patient‐derived organoids were established (Figure 3l), and a significant decreased Erastin‐induced ferroptosis in hMCs was observed when co‐cultured with organoid hMCs (Figure 3m).

AKT pathway was significantly enriched (Figure 2h) and confirmed (Figure S4h, Supporting Information) in TAMCs, which have been reported as regulator for ferroptosis process.^[^ ^21^ ^]^ We suspected that tumor conditioned medium mediated resistance to ferroptosis in TAMCs was AKT pathway dependent. As expected, the inhibitors of AKT pathway eliminated the protective effect of KPC‐conditioned medium (Figure 3n) and Panc02‐conditioned medium (Figure S4i, j, Supporting Information). The expression of GPX4 and SLC7A11 was also restored after exposure to AKT inhibition, suggesting that AKT activation mediated by the conditioned medium participated in the ferroptosis process of MCs (Figure 3o). Collectively, our findings suggest that PDAC cell‐derived exosomes inhibit cell death by preventing the ferroptosis cascade.

### 2.4 Sodium Cromoglycate Abrogates PDAC Progression Fostered by Tumor‐Associated Mast Cell Derived CXCL10

**Figure 4:**
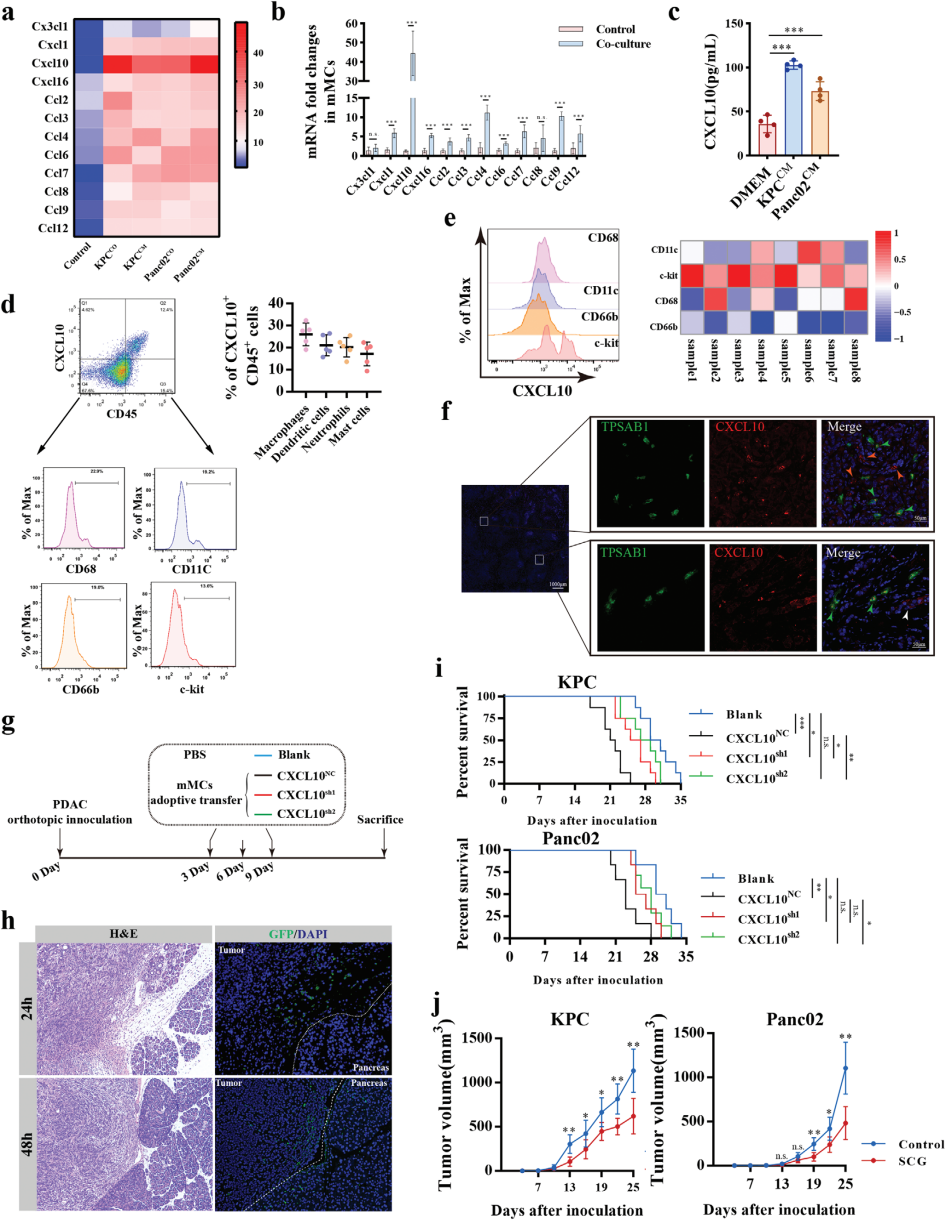
Tumor associated mast cell‐derived CXCL10 promotes tumor progression. a) Heatmap showing the significant changes of cytokines in both KPC and Panc02 educated mast cells by RNA‐seq. Mast cells were co‐cultured with tumor cells or stimulated by tumor‐conditioned medium for 24 hours. b) qRT‐PCR analysis for indicated cytokine transcriptions in mast cells that co‐cultured with KPC cells or not for 24 h. n = 4 per group. c) ELISA assay analysis for CXCL10 levels in mMCs supernatant after mMCs stimulated by indicated tumor‐conditioned medium. n = 4 per group. d) Flow cytometry analysis for CXCL10 expression in tumor‐infiltrating immune cells. The cell suspension was first gated by single cells and then CD45^+^CXCL10^+^ double positive cells were further divided into four types of immune cells according to the corresponding representative markers. CD68, macrophages; CD11c, dendritic cells; CD66b, neutrophils; c‐kit, mast cells. e) Flow cytometry analysis for CXCL10 expression in tumor‐infiltrating macrophages, dendritic cells, neutrophils and mast cells in PDAC tissues. The representative histogram (left) and relative normalized MFI expression (right) in four types of tumor‐infiltrating immune cells were displayed. f) Representative immunofluorescence staining of CXCL10 and TPSAB1 in PDAC tissues. TPSAB1, green; CXCL10, red; DAPI, blue. Green arrow: TAMCs; white arrow: tumor cells; orange arrow: other cells with CXCL10 positive expression. g) Schematic representation of mMC adoptive transfer in murine models. Blank: without MCs adoptive transfer; CXCL10^NC^: adoptive transfer MCs with negative control plasmid transfection; CXCL10^sh1^ and CXCL10^sh2^: adoptive transfer MCs with CXCL10 knockdown plasmid 1 and plasmid 2 transfection. h) Representative H&E staining and immunofluorescence staining for tumor‐infiltrating engineered mMCs labeled by GFP. The tumor tissues were collected after mMCs adoptive transferred for 24 or 48 hours. i) Kaplan‐Meier analysis of survival for orthotopic PDAC mice received mMCs adoptive transfer or not. Survival curves were analyzed by log‐rank tests. n = 8 per group. j) Tumor growth curves in KPC (left) and Panc02 (right) subcutaneous PDAC models with SCG intraperitoneal injection or PBS as control in C57BL/6. Student's t test was used for comparison. n = 6 per group. n.s. *P* > 0.05, *P < 0.05, **P < 0.01, and ***P < 0.001, Data were displayed as mean ± SD.

RNA‐seq analysis identified twelve cytokines that exhibited notably changed in TAMCs compared with MCs (**Figure** **4**a) and validated by qRT‐PCRs (Figure 4b). Notably, *cxcl10* mRNA showed the highest variation after co‐culturing and was the only prognostic indicator among them (Figure S5a, Supporting Information), suggesting the potential role of TAMCs‐derived CXCL10 in promoting tumor progression. The ELISA analysis revealed a significant increase of CXCL10 secretion by mMCs upon culturing with PDAC conditioned medium (Figure 4c). Concomitantly, the expression of *cxcl10* in PDAC was significantly higher compared to their adjacent normal tissues (Figure S5b, c, Supporting Information). CXCL10 was significantly associated with shorter OS in PDAC patients from the TCGA cohort (1.09 years versus 1.81 years, P < 0.001, Figure S5d, Supporting Information) and CTPAC cohort (0.95 year versus 2.18 years, P < 0.001, Figure S5e, Supporting Information). Among tumor‐infiltrating immune cells, TAMCs are identified as an important source of CXCL10 in PDAC tissues by flow cytometry (Figure 4d,e), and immunofluorescence staining (Figure 4f).

To investigate the impact of TAMC‐derived CXCL10 in vivo, we constructed CXCL10 knockdown mMCs with GFP overexpression (Figure S6a, Supporting Information) and validated it (Figure S6b, Supporting Information). The gene‐manipulated mMCs were then adoptively transferred to an orthotopic murine PDAC model (Figure 4g). After adoptive transfer, the mMCs primarily distributed to peripheral tumor tissues within 24 h and infiltrated into tumor lesions at 48 h (Figure 4h), confirming the recruitment of circulating MCs into the PDAC TME in vivo. Mice transplanted with CXCL10^sh^ mMCs had significantly reduced tumor burden (Figure S6c, d, Supporting Information) and prolonged survival compared to those transplanted with CXCL10^NC^ mMCs (Figure 4i), highlighting the pro‐tumoral role of TAMCs‐derived CXCL10 in vivo.

It has been reported that sodium cromoglicate (SCG) can inhibit the degranulation and cytokines release in MCs.^[^ ^8^, ^22^ ^]^ In this study, we found that SCG could also decrease CXCL10 secretion in vitro, suggesting a therapeutic effect by inhibiting mMCs function (Figure S6e, Supporting Information). As expected, SCG administration significantly restrain tumor growth (Figure 4j; Figure S6f, g, Supporting Information). Notably, SCG did not directly affect cancer cell proliferation (Figure S6h, Supporting Information) and CXCL10 secretion (Figure S6i, Supporting Information) in vitro, suggesting that the therapeutic effect of SCG dependent on inhibiting functions of TAMCs. Together, our observations support the idea that TAMCs promote PDAC progression through the secretion of CXCL10, and this process can be abrogated by SCG.

### 2.5 Tumor‐Derived Exosome miR‐188‐5p Promotes CXCL10 Transcription via PTEN/AKT/ERG Axis

**Figure 5:**
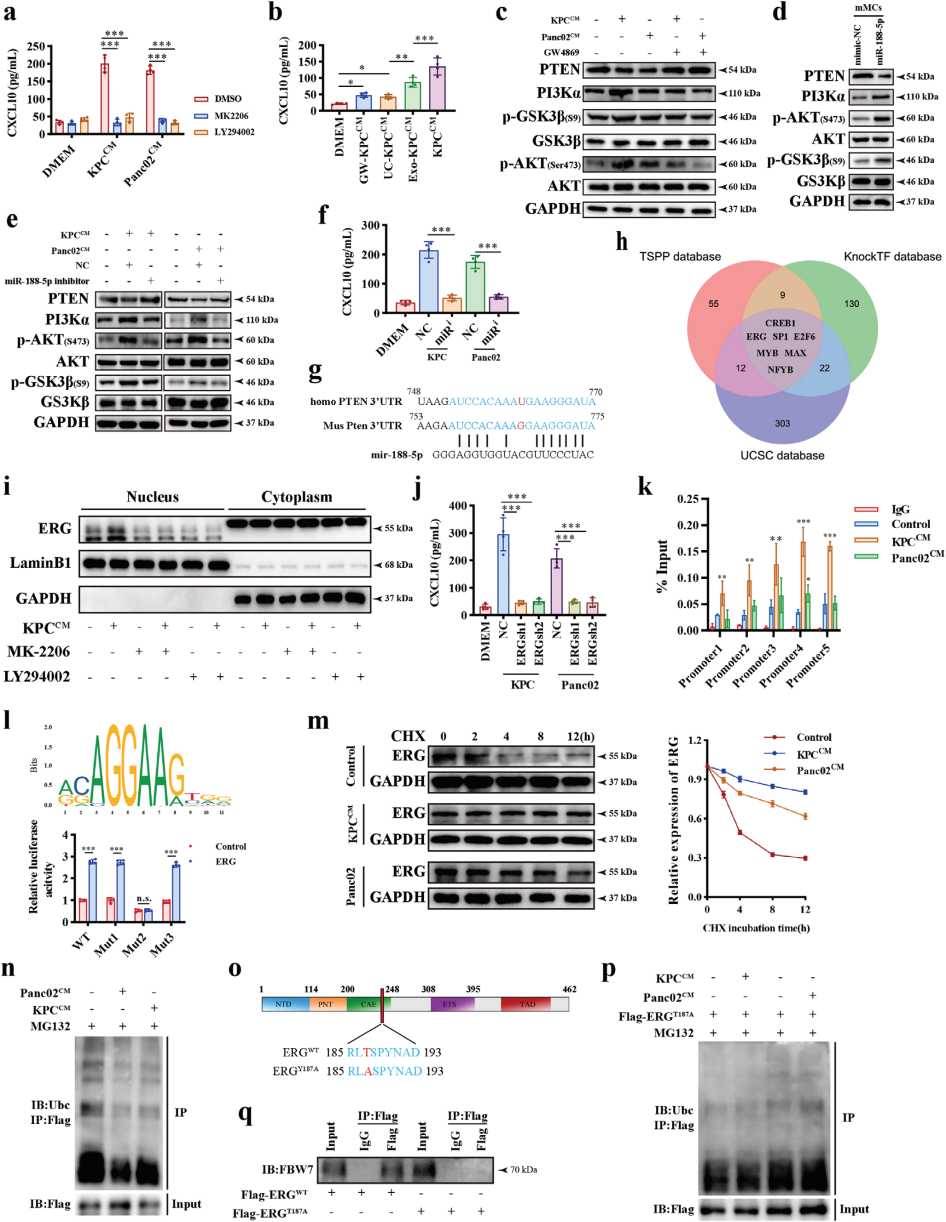
Tumor‐derived exosome miR‐188‐5p promotes CXCL10 transcription via PTEN/AKT/ERG axis in tumor‐associated mast cells. a) ELISA assay analysis for supernatant CXCL10 levels from tumor‐associated mast cells with or without AKT inhibitor pretreatment. n = 4 per group. b) ELISA assay analysis for supernatant CXCL10 level from tumor‐associated mast cells stimulated by conditioned medium with or without exosomes. n = 4 per group. c) Western blot analysis for AKT pathway activation in mast cells stimulated by normal or exosomes‐depleted KPC or Panc02 conditioned medium for 24 h. d) Western blot analysis for AKT pathway activation in tumor‐associated mast cells after miR‐188‐5p transfected. e) Western blot analysis for AKT pathway activation in tumor‐associated mast cells. KPC were transfected with miR‐188‐5p or negative control for 48h, and then the conditioned medium was harvested and used for mast cells stimulation. f) ELISA assay analysis for CXCL10 levels in tumor‐associated mast cell supernatant after indicated conditioned medium stimulation. n = 4 per group. miR^i^, miR‐188‐5p inhibitor. NC, negative control. g) The predicted binding site of miR‐188‐5p and PTEN 3′UTR region. h) Integrated bioinformatics analysis for potential transcriptional factors (TF) that could bind to Cxcl10 promoter. TSPP (https://www.signalingpathways.org/index.jsf) and UCSC (https://genome.ucsc.edu/) database were used for predicting TF binding to cxcl10 promoter. KnockTF (https://bio.liclab.net/KnockTF/search.php) were used for analyzing activated TF via different gene expressions. i) Western blot analysis for ERG nuclear translocation in tumor‐associated mast cells following AKT pathway inhibitor. j) ELISA assay analysis for CXCL10 levels in tumor‐associated mast cell supernatant after ERG knockdown by si‐RNA. n = 4 per group. k) ChIP‐PCR assay for ERG binding sites to Cxcl10 promoter following indicated treatments. n = 3 per group. l) Schematic representation of ERG binding sites in JARSPAR database (upper) and dual‐luciferase reported assay (lower) for binding sites identification. Student's t test was used for comparison. n = 4 per group. The difference of three mutated plasmids is described in Supplementary Figure 9D. m) Western blot analysis for the effect of tumor‐conditioned medium on the half‐life of ERG in tumor‐associated mast cells treated with CHX (100µg mL^−1^) for indicated incubation time. n) Western blot analysis for the effect of KPC and Panc02 conditioned medium on the ubiquitination level of ERG in mMCs. o) Schematic of the domain structure of ERG and the mutated site. p) The ubiquitination level of ERG T187A mutated protein after tumor‐conditioned medium stimulation in the presence of MG132 in mMCs. q) Western blot analysis for IP enriched cell lysates from mMCs. mMCs were transfected with Flag‐ERG^WT^, Flag‐ERG^T187A^ for 48 hours and lysed for co‐IP assay. ERG was enriched by Flag antibody or isotype IgG as control. n.s. *P* > 0.05, *P < 0.05, **P < 0.01, and ***P < 0.001, Data were displayed as mean ± SD.

A significant upregulation of *cxcl10* mRNA in TAMCs, indicating that CXCL10 was regulated at the transcriptional level. We found that AKT inhibition effectively resorted CXCL10 transcription in TAMCs (**Figure** **5**a; Figure S7a, Supporting Information), Furthermore, tumor‐conditioned medium without exosomes not only suppressed the transcriptional activity of CXCL10 (Figure 5b; Figure S7b, c, Supporting Information), but also significantly decreased AKT activation (Figure 5c).

Non‐coding RNA can be packaged into exosomes and affect target cells behavior. We enriched the target genes of differentially expressed miRNA in exosomes from KPC and murine pancreatic ductal cell (MPDC) as previously reported ^[^ ^23^ ^]^ and found PI3K‐AKT pathway as prominently enriched pathway in this dataset (Figure S7d, Supporting Information). Notably, we found a significant decrease expression of PTEN, an important negative regulator for AKT activation, after co‐culturing with PDAC cells, suggesting that it could be the mechanism underlying AKT pathway activation in TAMCs (Figure 5c). Subsequently, we screened out three miRNAs as potential candidates that were significantly upregulated in KPC exosomes and targeted PTEN. Among them, miR‐188‐5p was remarkably upregulated in TAMCs (Figure S7e, Supporting Information), and overexpression of miR‐188‐5p in MCs activated AKT pathway (Figure 5d). Conversely, conditioned medium from tumor treated with a miR‐188‐5p inhibitor reversed the phosphorylation of AKT in mMCs (Figure 5e), and also restored CXCL10 secretion in TAMCs (Figure 5f; Figure S7f, Supporting Information). Furthermore, we identified a highly conserved nucleotide binding site of miR‐188‐5p in PTEN (Figure 5g). The interaction of miR‐188‐5p and PTEN was confirmed in a luciferase assay, which has been reported in renal and vascular disease,^[^ ^24^ ^]^ further suggesting that miR‐188‐5p could be the key molecule responsible for AKT activation in TAMCs. Collectively, these results demonstrate that tumor‐derived exosome miR‐188‐5p promotes AKT activation in TAMCs by degrading PTEN.

To further explore the transcriptional factors (TFs) that mediated this process, seven potential candidates were screened out by overlapping the TF predicted by UCSC, TSPP and knockTF database (Figure 5h). Subsequently, the correlations between TFs and CXCL10 in pan‐cancer datasets were analyzed. Top Five TFs with the closest correlation with CXCL10 were chosen as candidates and knocked down for further studies (Figure S8a, b, Supporting Information). Among them, ERG and SP1 knockdown was found to be significantly effective in downregulating CXCL10 expression in MCs (Figure S9a, Supporting Information).

ERG significantly translocated into nuclear after stimulation with tumor‐conditioned medium, which was reversed by AKT pathway inhibitors in mMCs (Figure 5i; Figure S9b, Supporting Information). However, the nuclear translocation of SP1 showed no significant change (Figure S9c, Supporting Information). Importantly, ERG knockdown effectively restored CXCL10 secretion in MCs (Figure 5j). Additionally, the ChIP assay showed that ERG could bind to the CXCL10 promoter (Figure 5k). Mutated CXCL10 luciferase promoter plasmids were constructed to further validate the binding site between ERG and CXCL10 (Figure 5l; Figure S9d, Supporting Information). Collectively, these results demonstrate that PDAC‐derived exosome miR‐188‐5p facilitates CXCL10 transcription through the PTEN/AKT/ERG axis.

We found that tumor‐conditioned medium did not affect transcriptional expression (Figure S9e, Supporting Information), but significantly increased ERG protein level (Figure S9f, Supporting Information). This suggested that AKT may regulate ERG through post‐transcriptional modifications (PTMs). Cycloheximide inhibition assay revealed that tumor‐conditioned medium prolonged half‐life of ERG (Figure 5m), indicating that PTM‐mediated ERG accumulation in MCs is a key mechanism underlying the upregulation of CXCL10. As ubiquitination is an important mechanism in protein degradation, we analyzed the ubiquitin level of ERG and found that tumor supernatant decreased ERG ubiquitination (Figure 5n). A previous study reported that GSK3β could phosphorylate ERG^T187^, thereby promoting its interaction with FBW7, an important E3 ubiquitin ligase.^[^ ^25^ ^]^ We demonstrated a significant increase in the phosphorylation of GSK3β following treatment with tumor‐conditioned medium (Figure 5c), indicating that GSK3β might play a similar role in inhibiting ERG ubiquitination in TAMCs. Furthermore, GSK3β knockdown in TAMCs could reserve the effect of tumor‐conditioned medium on ERG ubiquitination levels (Figure S9g, Supporting Information).

Next, the 3D structure of the mERG protein was analyzed (Figure S9h, Supporting Information), and mut‐mERG^T187A^ plasmid were constructed as previously reported (Figure 5o).^[^ ^25^ ^]^ Notably, mut‐ERG^T187A^ plasmid abrogated the stabilizing effect of tumor‐conditioned medium (Figure 5p). Furthermore, the ERG mutation impaired the interaction between GSK3β‐ERG‐FBW7 complex (Figure 5q), indicating that FBW7 interacted with ERG and promoted its ubiquitination, depending on the phosphorylation of Thr^187^. These results revealed that tumor‐conditioned medium increased ERG expression via PTMs, promoted ERG nuclear translocation, bind to the CXCL10 promoter, and ultimately resulted in the upregulation of CXCL10 transcription.

### 2.6 Tumor‐Associated Mast Cell‐Derived CXCL10 Promotes PDAC Cell Mobility and Stemness via CXCR3‐PPARγ Axis

**Figure 6:**
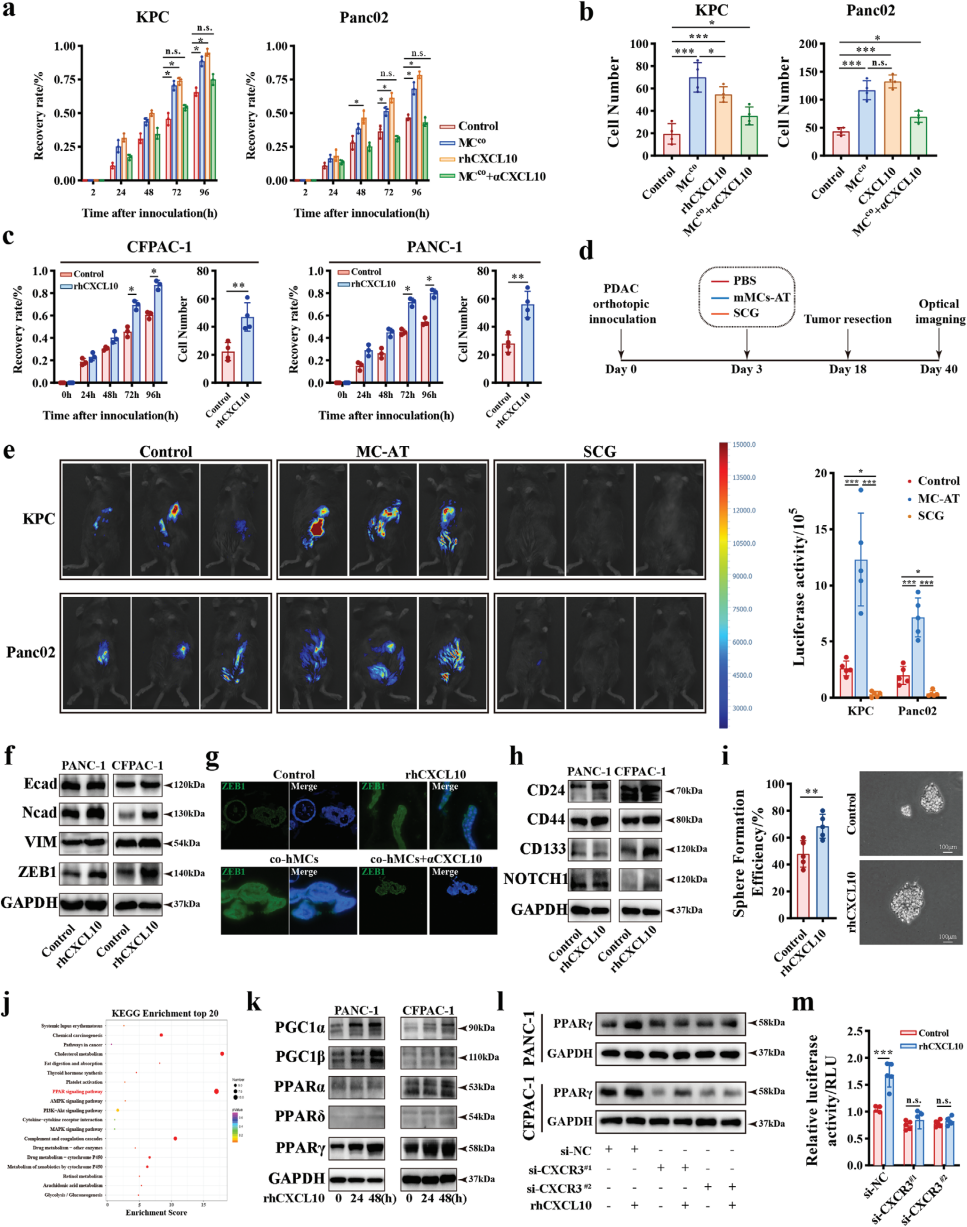
CXCL10 activates PDAC tumor migration and stemness by activating PPARγ. a) and b) Wounding assay (a) and transwell assay (b) analysis for KPC and Panc02 migration ability by co‐culturing with mast cell for 24 h associated with CXCL10 antibody or isotype IgG as control, or stimulating with rmCXCL10 (100ng mL^−1^) for 24 h. n = 4 per group. c) Quantification of migration ability by wounding scratch assay (n = 3) and transwell assay (n = 4) in CFPAC‐1 (left) and PANC‐1 (right) stimulated by rhCXCL10 (100ng mL^−1^) or not. d) Schematic for KPC and Panc02 orthotopic tumor models that received tumor surgical resection after mMCs adoptive transfer or SCG treatment. Recurrence or metastasis after surgical resection were evaluated by IVIS imaging system. e) Representative optical image in KPC and Panc02 murine models (left) with different treatments, and quantification analysis (right) of luciferase optical density. n = 5 per group. f) Western blot analysis for EMT‐related proteins in cancer cells treated with rhCXCL10 for 24 h. g) The representative image of immunofluorescence staining for ZEB1 in PDAC patient‐derived organoid slices after indicated treatments. h) Western blot analysis for stemness markers in cancer cells treated with rhCXCL10 for 24 h. i) Sphere formation assay analysis for PANC‐1 pre‐treated with rhCXCL10 or control for 24 h. n = 5 per group. j) KEGG functional enrichment analysis of DEGs between PANC‐1 stimulated by rhCXCL10 or not for 24 h. k) Western blot analysis for PPAR pathway in PANC‐1 and CFPAC‐1 stimulated by rhCXCL10 or not. l) Western blot analysis for PPARγ expression in the indicated cells. CXCR3 were knockdown by siRNA transfection in PANC‐1 and CFPAC‐1, and then stimulated by rhCXCL10 for 24 h. m) Dual‐luciferase assay analysis for PPAR transcriptional activity in PANC‐1 and CFPAC‐1 after rhCXCL10 stimulation. n = 5 per group. n.s. *P* > 0.05, *P < 0.05, **P < 0.01, and ***P < 0.001, Data were displayed as mean ± SD.

CXCL10, also known as interferon‐inducible protein 10, interacts with CXCR3 and selectively attracts CXCR3^+^T cells into the TME.^[^ ^26^ ^]^ CXCL10 could also impact tumor growth, apoptosis and metastasis by targeting CXCR3 on tumor cells.^[^ ^27^ ^]^ To decipher the mechanism through which CXCL10 promotes malignant behavior in PDAC, we co‐cultured MCs with murine PDAC cells, and observed that TAMCs co‐culturing and rmCXCL10 stimulation could obviously promote tumor migration and invasion (**Figure** **6**a,b; Figure S10a‐c, Supporting Information). However, no proliferation effect was observed after TAMCs treatment (Figure S10d, Supporting Information), indicating that TAMCs indirectly promote tumor growth in vivo.

Similarly, CXCL10 does not directly affect tumor growth and apoptosis in vitro (Figure S11a‐e, Supporting Information). However, rhCXCL10 stimulation significantly enhanced tumor migration (Figure 6c; Figure S12a, b, Supporting Information) and invasion (Figure S12c, Supporting Information). CXCL10 overexpression in PDAC cells also increased liver metastasis tumor burden in vivo (Figure S12d, Supporting Information). The association between CXCL10 and epithelial‐mesenchymal transition (EMT) or metastatic signatures were also identified in the TCGA database (Figure S12e, Supporting Information). Furthermore, EMT‐related genes were significantly increased after CXCL10 stimulation (Figure S12f, Supporting Information).

Then, an orthotopic PDAC murine model was established and surgical resection for tumors was performed on Day 18 (Figure 6d). Adoptive transfer of mMCs significantly promoted distant metastasis and local recurrence after surgical resection, while SCG treatment significantly inhibited distant metastasis (Figure 6e; Figure S12g, Supporting Information). Tumor cells invaded into adjacent pancreas in MCs‐AT group in resected tissues (Figure S12h, Supporting Information). The RNA‐seq was also performed for PANC‐1 with or without CXCL10 stimulation, and showed that rhCXCL10 stimulation significantly upregulated N‐cadherin, Vimentin, and ZEB1 expression, coinciding with increasing mobility following rhCXCL10 stimulation (Figure S13a, Supporting Information). Similar changes were observed in human PDAC cells (Figure 6f). In patient‐derived organoids upon rhCXCL10 stimulation or coculturing with hMCs, ZEB1 was similarly obviously upregulated and underwent nuclear translocation (Figure 6g). The association between CXCL10 and the stemness signature was also observed in the TCGA database (Figure S13b, Supporting Information). rhCXCL10 was found to upregulate stemness markers and spheres formation ability in vitro (Figure 6h,i; Figure S13c, d, Supporting Information). Immunohistochemistry also validated the upregulated expression of EMT and stemness markers in murine orthotropic tumor tissues (Figure S13e, Supporting Information). These results collectively indicate that CXCL10 can promote the mobility and stemness of PDAC cells.

To further explore the underlying mechanism by which CXCL10 mediates mobility, KEGG pathway enrichment analysis was performed, and identified that the PPAR pathway was significantly enriched in the rhCXCL10 stimulated group (Figure 6j). PPARγ and its co‐activators PGC1α and PGC1β were significantly upregulated after CXCL10 stimulation (Figure 6k; Figure S14a, Supporting Information). Intrinsic PPARγ knockdown reversed CXCL10‐mediated changes of EMT makers and migration ability (Figure S14b‐d, Supporting Information).

CXCR3 has also been found in tumor cells (Figure S14e, Supporting Information), leading to the speculation that PPARγ may be activated following CXCL10‐CXCR3 stimulation. CXCR3 knockdown was found to significantly decrease the effect of CXCL10 on PPARγ (Figure 6l; Figure S14f, Supporting Information). Additionally, CXCR3 knockdown obviously reduced the translational activity of the PPAR luciferase report plasmid after CXCL10 stimulation (Figure 6m; Figure S14g, Supporting Information). Finally, the PPARγ knockdown significantly reversed the stemness markers and the capacity of sphere formation induced by CXCL10 stimulation (Figure S14h, i, Supporting Information). Overall, these results demonstrate that CXCL10 can promote the mobility and stemness in PDAC through CXCR3/PPARγ axis.

### 2.7 Blockade of Tumor‐Associated Mast Cell‐Derived CXCL10 as a Strategy by Reducing CXCR3^+^Tregs Mediated Immune Escape

**Figure 7:**
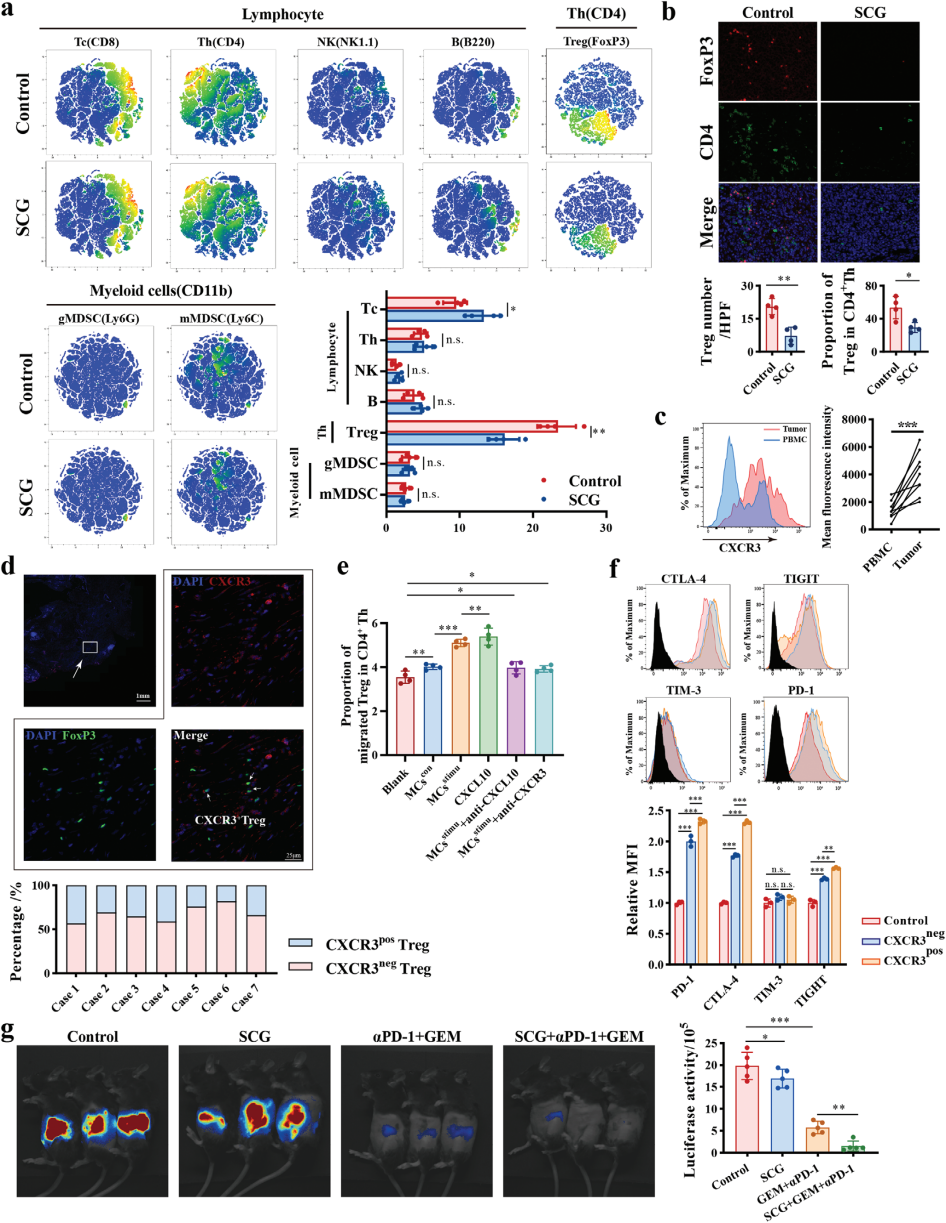
CXCL10 recruits CXCR3^+^ Tregs into microenvironment and SCG synergizes with gemcitabine plus αPD‐1. a) TSNE analysis for tumor‐infiltrating immune cells with or without SCG treatment and the proportions of these cells. KPC cell lines were orthotopically inoculated in C57BL/6 mice and received SCG treatment, tumor tissues were harvested at 20 days and analyzed by flow cytometry (n = 4). b) Dual‐fluorescence staining images and quantification of tumor‐infiltrating Treg in SCG treatment and control group. Foxp3, red; CD4, green; DAPI, blue. c) Flow cytometry analysis (left) and MFI quantitative analysis (right) of the CXCR3^+^ level on Treg in paired PMBCs and PDAC tissues. d) Dual‐fluorescence staining of CXCR3^+^ Treg in PDAC tissues (upper). CXCR3, red; FoxP3, green; DAPI, bule. The percentage of CXCR3^+^Treg in total tumor infiltrated Treg were calculated in seven PDAC patients (lower). e) Quantification of Treg proportions in migrated CD4^+^ cells recruited by mMCs‐conditioned medium with or without antibodies blockade in migration assay. Murine spleen was dissociated as single cells and added into upper chamber, indicated medium were then added into lower chamber for migration assay. n = 4 per group. f) Flow cytometry analysis for the level of exhausted markers in T cells when coculturing with CXCR3^+^ or CXCR3^−^ Treg sorted by FACS. n = 3 per group. g) SCG was intraperitoneal injection at 75mg kg^−1^ for three times per week. Gemcitabine (20mg kg^−1^) plus αPD‐1 (100µg per mouse) were intraperitoneal injection once a week. 20 days after inoculation, luciferase activity was detected by IVIS imaging. Representative optical image (left) in KPC murine models and quantification (right) of luciferase optical density. n = 5 per group. n.s. *P* > 0.05, *P < 0.05, **P < 0.01, and ***P < 0.001, Data were displayed as mean ± SD.

To further investigate the effect of SCG on immune microenvironment, we examined tumor‐infiltrating immune cells and found a significant decrease in Tregs after SCG treatment by flow cytometry and immunofluorescence staining (**Figure** **7**a,b). It is widely accepted that Th1 cells preferentially express CXCR3 and CCR5, while CCR8 and CCR4 are highly expressed in Th2 and FoxP3^+^Tregs. However, a specific subset of CXCR3^+^Treg has been identified in cancers and known to promote tumor progression.^[^ ^28^ ^]^ This specific subtype may be attracted by CXCL10, which supports our hypothesis that SCG can influence TME by inhibiting the secretion of CXCL10 from TAMCs. We also found a higher proportion of CXCR3^+^Tregs in tumor tissues compared to PBMCs, indicating that CXCR3 expression on Tregs plays a vital role in their chemotaxis (Figure 7c). The presence and percentage of CXCR3^+^Tregs was further confirmed in human PDAC tissues through immunohistochemistry (Figure 7d). We found that MCs induced the migration of CXCR3^+^ Tregs in a CXCL10‐dependent manner in chemotaxis assay (Figure 7e). Co‐culturing T cells with or without CXCR3^+^/CXCR3^−^Tregs isolated from peripheral PBMC revealed that CXCR3^+^Tregs could also inhibit CD8^+^T cell function (Figure 7f). Therefore, TAMCs can attract CXCR3^+^Tregs into TME, and thereby induce a stronger immunosuppressive response in PDAC.

Chemo‐immunotherapy revolutionizes cancer treatment and holds promise for PDAC.^[^ ^29^ ^]^ As our preceding results, TAMCs‐derived CXCL10 recruits CXCR3^+^Treg into TME, resulting in increasing exhausted phenotypes in CD8^+^T cells, notably PD‐1, in PDAC. This indicates that αPD‐1 may also restore cytotoxic T cells exhaustion induced by TAMCs. Additionally, stemness of cancer cells is a key factor in gemcitabine resistance, suggesting that blocking CXCL10 from TAMCs by SCG could also be a potential combination to enhance chemotherapeutic efficacy. Therefore, we hypothesized that SCG might provide a synergistic effect when combined with gemcitabine and αPD‐1.

In orthotopic murine PDAC model, we found that SCG alone slightly inhibited tumor growth and the combination of SCG with gemcitabine/αPD‐1 was more effective than each agent alone (Figure 7g). These results indicate that SCG sensitize the therapeutic efficacy of gemcitabine plus αPD‐1 in PDAC and could be a promising adjuvant for chemo‐immunotherapy in PDAC.

## 3 Discussion

Complex communication networks between tumor and immune cells play a critical role in tumor progression and drug resistance. This study demonstrates that a crosstalk between PDAC cells and TAMCs ultimately contributes to cancer metastasis and immuno‐chemotherapeutic resistance, as illustrated in Figure S15 (Supporting Information). In light of these findings, we propose SCG as a promising therapeutic strategy by inhibiting MCs’ CXCL10 secretion, disrupting the crosstalk between MCs and tumor cells, and thereby alleviating tumor metastasis while enhancing sensitivity to immuno‐chemotherapy.

CXCR2 is vital for the recruitment of MCs in physiological ^[^ ^30^ ^]^ and pathological conditions.^[^ ^31^ ^]^ In this study, we have also demonstrated that CXCR2 plays a crucial role in recruiting MCs in PDAC, while CXCR4 is the main chemotaxis receptor for MC infiltration in gastric cancer.^[^ ^16^ ^]^ This highlights the diversity of mast cells in various cancers. Furthermore, TAMCs in gastric cancer rarely expressed Ki‐67, which is in stark contrast to PDAC, where the majority of TAMCs (>85%) were Ki‐67 positive in our study.

Ferroptosis is a non‐apoptotic, iron‐dependent form of programmed cell death characterized by lipid peroxide accumulation. Our study demonstrated that upregulated GPX4 and SLC7A11 is consistent with decreased ferroptosis in TAMCs after tumor conditioned medium stimulation. However, ACLS4 was reversely upregulated after treatment with tumor‐conditioned medium, which was contradicted to ferroptosis resistance in TAMCs. The Hippo pathway, especially the YAP‐TEAD complex, has been reported to bind to the ACSL4 promoter.^[^ ^32^ ^]^ Our data also revealed a significant enrichment of the Hippo as the second enriched pathway in TAMCs, which may explain the aberrant high expression of ACSL4 in low ferroptosis level. Ferroptosis sensitivity is determined by the susceptibility to lipid peroxidation and the overall defense capacity of cells.^[^ ^33^ ^]^ TAMCs exhibited significantly high lipid ROS generation and elimination, while antioxidative process through GSH/GSSH had higher activity, counteracting the effect of ACSL4.

However, it is worth to note that treatment with tumor‐conditioned medium restored RSL3‐induced ferroptosis in MCs, suggesting that the inhibition of ferroptosis in MCs is not solely mediated by System Xc‐ and GPX4‐dependent mechanisms. It had been reported that purified GPX4 alone cannot be inhibited by RSL3, indicating the involvement of other cellular components in this process.^[^ ^34^ ^]^ Tumor conditioned medium treatment could interrupt the interaction and rescue cells from ferroptosis. On the other hand, ferroptosis occurs after enough lipid peroxidation accumulation. Sebastian et.al reported that cells lacking GPX4 activity showed ferroptosis resistance if lipid ROS production is disrupted at the same time.^[^ ^35^ ^]^ Fe^2+^ ion is crucial for lipid peroxide by Fenton reaction. Labile iron pool (LIP) can contribute to spontaneous oxidation and further drive ferroptosis.^[^ ^36^ ^]^ We observed significant decrease of Fe^2+^ in TAMCs, which could be other potential mechanism that contribute to GPX4‐independent ferroptosis. Thus, it is worth to further investigate the details of how PDAC inhibits ferroptosis in MCs and alleviates the accumulation of MCs by targeting potential mechanisms.

MCs have transcriptional diversity depending on different surroundings. One classification for MCs is based on their produced proteases, by categorizing as tryptase only (MC_T_), chymase only (MC_C_), or a combination of both (MC_TC_).^[^ ^37^ ^]^ In this scenario, MCs could induce various biological function when releasing the proteases. However, we found an impaired degranulation capacity in TAMCs compared with MCs, highlighting chemokine/cytokine secretion is of great significance in TAMCs. Secreted histamine is the key molecular in anaphylactic reactions, and this could inhibit PDAC cells growth in vitro.^[^ ^38^ ^]^ In tumor microenvironment, the subtype of histamine receptors (HRs) differential expression and different intracellular signaling cause the diversity biological function. HR1‐2 are both expressed endothelial cells, which participate in histamine mediated vasodilation and increase vascular permeability.^[^ ^39^ ^]^ H3R is expressed in the central nervous system. H4R has a stronger capacity in response to histamine, IgE, and compound 48/80 than that observed with H1R antagonists.^[^ ^40^ ^]^ In PDAC, TAMCs showed decreased secretion of histamine, so we paid attention to its cytokine secretion. In this study, we identified CXCL10 as crucial chemokine in TAMCs. CXCL10 is the ligand for CXCR3, which have three variants including CXCR3a, CXCR3b and CXCR3‐alt, with CXCR3a binding to CXCL9, CXCL10, and CXCL11, while CXCR3b preferably binds to CXCL4.^[^ ^41^ ^]^ Our results showed that significant CXCL10 upregulation in TAMCs compared with MCs. There are still some controversies of CXCL10 biological function in tumors. High CXCL10 expression in tumor tissue correlated with poor survival in PDAC, even though CXCL10 exhibited anti‐tumor effect by recruiting CXCR3 positive immune cells to remodel TME, including CXCR3^+^CD4^+^T cells and CXCR3^+^CD8^+^T cells.^[^ ^42^ ^]^ In our study, we found notably CD8^+^T cells increase after SCG treatment in vitro, contradictorily with decreasing TAMCs‐derived CXCL10 secretion. We suspected that this was caused by synchronous recruitment of CXCR3^+^Tregs. CXCR3^+^Treg is a strong immunosuppressive subtype, which could suppress T cells function by both contact‐dependent^[^ ^28^ ^]^ and independent^[^ ^43^ ^]^ way. After SCG treatment, decreased TAMCs‐derived CXCL10 recruited less CXCR3^+^Treg into tumor, and this might be one possible reason that caused the increasing CD8^+^T cells infiltration in our study. In addition, CXCR3^+^Treg also significantly inhibit effective T cells proliferation,^[^ ^44^ ^]^ this might be another factor that less CXCR3^+^Treg infiltration promote more CD8^+^T cells proliferation and finally led to CD8^+^T cells accumulation. Therefore, the balance between CXCR3^+^CD8^+^ cells and CXCR3^+^Tregs decided the effect of CXCL10 on TME. Additionally, peripheral blood mononuclear cells of PDAC patients contained more Tregs, indicating that circulating Tregs could be preferentially recruited by CXCL10 compared to other cells.^[^ ^45^ ^]^

The issues of recurrence and delayed distant metastasis following radical tumor resection cannot be overlooked in PDAC. Our findings indicated that TAMCs increased the recurrence rate in PDAC and led to shorter OS and RFS. We found that TAMCs could promote PDAC cells EMT transition by targeting tumoral CXCR3, which was responsible for tumor metastasis. Similar observation has been reported in thyroid cancer.^[^ ^46^ ^]^ CXCL10 activated tumoral CXCR3 and its downstream in PDAC cells, finally promoted ZEB1 nuclear translocation. ZEB1 mediated EMT transition is closely associated with the acquisition of stemness in tumor cells, and implicated in resistance to gemcitabine‐based chemotherapy.^[^ ^47^ ^]^ Currently, gemcitabine is regarded as the first‐line treatment for advanced PDAC. One clinical trial in metastatic PDAC observed modest increases in OS with αPD‐1/GEM/Nab‐paclitaxel compared to chemotherapy alone.^[^ ^29^ ^]^ Moreover, in locally advanced PDAC, αPD‐1 antibody combined with chemotherapy significantly prolonged OS and PFS.^[^ ^48^ ^]^ Based on our findings, we conducted a preclinical trial using SCG as an adjuvant agent for immuno‐chemotherapy against PDAC, which yielded satisfactory outcomes.

In summary, our study clarifies the crosstalk between PDAC and TAMCs, which leads to the abnormal accumulation of TAMCs in tumor lesions via CXCR2‐mediated chemotaxis. Tumor‐derived exosome miR‐188‐5p promotes CXCL10 expression via PTEN/AKT/ERG signaling in TAMCs. Additionally, TAMCs‐derived CXCL10 reversely enhanced the mobility and stemness of PDAC cells via CXCR3/PPARγ axis, and recruits CXCR3+ Tregs into TME, thereby promoting tumor progression and immune escape. In pre‐clinical trials, SCG is confirmed as an effective agent in decreasing recurrence and dissemination after radical resection of PDAC, and significantly enhances sensitivity to current immuno‐chemotherapy. Therefore, SCG shows promise as a therapeutic adjuvant in the current strategy for PDAC.

However, this study had several limitations. First, we reported the ferroptosis protection of tumor conditioned medium for MCs, however, the underlying mechanism required further exploring. Second, the detailed mechanism of how CXCR3^+^Tregs inhibits CD8^+^T cell function in PDAC remains to be investigated. Third, even though negative correlation between TAMCs and survival has been reported in several studies, the clinical/pathological correlation with tumor‐infiltrating MCs level mains to be validated in another validation cohort.

In summary, this study identified the crosstalk between TAMCs and PDAC cells, and demonstrated an innovative mechanism that promotes PDAC progression and immunosuppression. CXCL10 blockade with SCG sensitizes PDAC to the current immuno‐chemotherapy, which reveals its potential to be a valuable adjuvant for PDAC patients.

## 4 Experimental Section

### Cell Cultures

*PDAC Cell Lines*: Human PDAC cell lines (PANC‐1 and CFPAC‐1) were purchased from the National Collection of Authenticated Cell Cultures and authenticated using STR profiles. Mouse‐derived PDAC cell lines, Panc02 and KPC cells (LSL‐Kras^G12D/+^; Trp53^R172H/+^; Pdx‐1^Cre/+^), were generously provided by Johns Hopkins Hospital. PANC‐1 and KPC cells were cultured in high glucose DMEM supplemented with 10% FBS; CFPAC‐1 cells were cultured in IMDM supplemented with 10% FBS; Panc02 cells were cultured in RPMI‐1640 supplemented with 10% FBS. All cell lines were maintained in a humidified atmosphere with 5% CO_2_ at 37 °C. Mycoplasma was routinely tested during cells culturing.

### Primary Immune Cells

For murine mast cells, progenitor mMCs were isolated and expanded from C57BL/6 femur bone marrow. Fresh bone marrow was incubated with RBC lysis buffer to lyse red blood cell, then filtered through 70µm sieve to remove cell lumps. The residual cells were maintained and amplified in RPMI‐1640 supplemented with IL‐3 (10ng mL^−1^), SCF (10ng mL^−1^) and 10% (v/v) FBS for 4 weeks. The purity was identified by flow cytometry and immunohistochemistry staining with indicated markers before further study.

Human CD34^+^ progenitor cells were enriched using an isolation kit from PBMC according to manufacturer's instructions, and differentiated into hMCs as previously reported.^[^ ^49^ ^]^ Human mast cells were stained with c‐kit and FcεR1a antibodies and identified the purity by flow cytometry.

Human T cells were isolated and amplified as previously reported.^[^ ^50^ ^]^ Briefly, PBMCs were isolated from peripheral blood and amplified in RPMI‐1640 containing anti‐CD3 (1µg mL^−1^), anti‐CD28 (1µg mL^−1^), and rhIL‐2 (10ng mL^−1^) and 10% (v/v) FBS.

Human Tregs from PBMCs were labeled by CD3^+^CD4^+^CD25^+^CD127^low^ and further sorted into CXCR3+ and CXCR3^low^ for CXCR3^pos^Treg or CXCR3^neg^Treg, respectively.

### PDAC Patient‐Derived Organoids

Three PDAC patient‐derived organoids were established for co‐culturing assay. In brief, surgical resected PDAC specimens from Zhongshan Hospital, Fudan University were digested by the Tumor Dissociation Kit, and embedded in Matrigel as dome. The domes were overlaid with organoid culture medium provided by Hangzhou Chexmed technology co., LTD. The medium was changed every 4 days. After the indicated treatment, the organoids were harvested using Organoid Harvest Solution and embedded in paraffin for organoid slice preparation.

### DNA/RNA and Protein Experiments

*Design and Construction for siRNA/shRNA, Luciferase‐Reported Plasmid, or miRNA Analogue/ Antagonist*: The siRNA targeting human SCF, PPARγ, and CXCR3 were purchased from Tsingke Biotechnology. Murine Creb1, Erg, Sp1, Max, and Nfyb si‐RNA were designed and synthesized by Yuanmin Biotech. miR‐188‐5p inhibitor and miR‐188‐5p mimic and negative control were purchased from RiboBio Co., Ltd. Lentivirus encoding murine *cxcl10*‐shRNA (CXCL10^sh1/sh2^) or negative control (CXCL10^NC^) and luciferase reported plasmid were designed and synthesized by GeneChem. Lentivirus was prepared by transfecting plasmid DNA and packaging plasmids in 293T cells, and virus supernatant was harvested after 48 h. The targeting sequences can be found in Table S3 (Supporting Information).

### Cytoplasm and Nuclear Separation, Western Blot, and Co‐Immunoprecipitation (co‐IP)

Cells suspensions lysed by RIPA for total protein analysis. Cytoplasmic and nuclear proteins were obtained using a cytoplasmic and nuclear separation kit. The expression of proteins in the cell lysate was assessed by western blot assays. Briefly, the proteins were transferred onto a PVDF membrane, and blocked with a 5% milk solution, and then incubated with indicated primary and secondary antibodies. The bands were visualized using an ECL imaging system.

For co‐IP, primary antibodies or IgG as negative control were incubated with Protein A/G Magnetic Beads for 30 minutes at room temperature and washed five times to remove unconjugated antibody. Then, cell lysates were incubated with conjugated antibodies for an additional 2 h at room temperature. The beads were then washed five times with washing buffer. To elute proteins for western blots, loading buffer were added into the beads, and the mixture was heated to 99 °C for 10 min to dissociate targeting proteins from beads. Finally, the proteins were used for western blot analysis.

### Enzyme‐Linked Immunosorbent Assay (ELISA)

The secretion of CXCL10 from TAMCs was evaluated using the Mouse CXCL10 Duoset ELISA kit, following the manufacturer's instructions. Mast cells were pretreated with tumor‐conditioned medium or DMEM as a control for 24 h. Afterwards, the cells were washed by PBS buffer to remove the initial medium, subsequently sed into fresh medium at concentration of 5×10^5^ mL^−1^ for 48 h, and finally indicated supernatants were collected for the ELISA analysis according to manufacturer's instruction. To eliminate evaporation effect on supernatant, the CXCL10 concentration in indicated supernatants was normalized to the final mast cells density as CXCL10^normalized^ = CXCL10^measurement^ × [cell density/5×10^5^]. The optical density of each group was measured at 450 nm using a microplate reader (Molecular Devices, China), and the concentration of CXCL10 was calculated using a standard curve.

### Immunohistochemistry and Immunofluorescence

Specimens were fixed in 4% paraformaldehyde, embedded in paraffin and sliced to 3µm sections. After deparaffinization, the slides for immunohistochemistry underwent antigen retrieval by microwaving, and non‐specific sites were blocked with 5% goat serum.

For immunohistochemistry, endogenous peroxidase was blocked using a 3% hydrogen peroxide solution. Slides were then incubated overnight at 4 °C with appropriate diluted primary antibodies, followed by incubation with a 1:500 diluted HRP‐conjugated secondary antibodies for an additional 2 h. The slides were stained by the BAD reagent and counterstained with hematoxylin. Staining intensity and density of positive cells were evaluated by two pathologists. The representative slides were captured by microscope (Olympus, Japan) or ScanScope XT scanner (Aperio Technologies). The density of TAMCs in PDAC were calculated by TAMCs (numbers) /stroma area (mm3) for each case. The expression of proteins for each sample was defined as the intensity of positive staining (brown).

For immunofluorescence, the slides were incubated overnight at 4 °C with appropriate diluted primary antibodies, followed by incubation with a fluorescence‐labeled secondary antibody for an additional 2 h. After washing out the secondary antibody, the slides were stained by DAPI. The representative images of indicated slices were captured by fluorescence microscope (Olympus, Japan). The evaluation methods of immunofluorescence staining were similar to immunohistochemistry.

### RT‐qPCR Assay and Chromatin Immunoprecipitation (ChIP)

The cells were lysed using RNAiso reagent, and total RNAs were extracted according to manufacturer's instructions. Then RNA was then reverse transcribed into cDNA using PrimeScript RT reagent kit. The indicated cDNA, primers, and SYBG Green Mix were mixed and analyzed using the QuantStudio™ 5 RT‐qPCR System (Applied Biosystems, USA). The expression of the target genes was normalized to GAPDH and quantified as ΔΔCt. The primer sequences used in this study are listed in Table S4 (Supporting Information).

The ChIP assay was performed using the Chromatin Immunoprecipitation Kit, following the manufacturer's instructions. Briefly, treated mast cells were cross‐linked by 1.5% formaldehyde and terminated by glycine. Nuclei were extracted from the collected cells and digested with micrococcal nuclease at 37 °C for 20 min. The resulting products were then incubated overnight at 4 °C with anti‐ERG antibody, IgG isotype as a control, and Histone H3 as a positive control. The lysates were subsequently incubated with Protein G beads at 4 °C for 2 h. The immunoprecipitation products were separated using a magnetic stand, and the DNA was extracted. The DNA was analyzed by qPCR, and the binding sites were evaluated based on the enrichment level. The primer sequences for the ChIP PCR assay can be found in Table S5 (Supporting Information).

### Clinical Data, Tumor Tissues, and Follow‐Ups

*Tissue Microarray and Follow‐Ups*: Tissue microarrays (TMA) were created using archival formalin‐fixed paraffin‐embedded tissue blocks obtained from surgically resected primary PDAC. This was done using a manual Tissue Puncher/Arrayer (Beecher Instruments, Silver Spring, MD) as previously described.^[^ ^51^ ^]^ We arrayed 102 cores with 1.4 mm diameters (containing 52 PDAC and 50 adjacent normal tissues) on recipient blocks at Johns Hopkins Hospital. Similarly, 172 cores (containing 90 PDAC and 82 adjacent normal tissues) from Zhongshan hospital were arrayed as TMAs for further analysis. The OS was calculated from the date of surgery to the time of death for any reason or to the time of censorship at the last follow‐up. The RFS was defined as the period between the date of surgery and the date of recurrence of disease or death, whichever occurred first or was censored at the last follow‐up. Informed consent was obtained from all patients, and this protocol was approved by the Ethics Committee of Zhongshan Hospital, Fudan University (No. B2023‐129).

### Infiltrating Immune Cell Analysis in PDAC

PDAC and its adjacent normal tissues were dissociated to single cell by the Tumor Dissociation Kit according to manufacturer's instructions. The single cells were collected and stained for surface markers at 4 °C for 30 min, and then fixed with IC Fixation Buffer. After washing three times by PBS buffer, the cell suspensions were permeabilized and stained for intracellular markers using Permeabilization Buffer. Finally, the data were acquired by the BD FACS Arial Flow Cytometer (BD Biosciences, USA), and analyzed using Flowjo V10 (BD Biosciences, USA).

### RNA Sequencing and Bioinformatic Analyses

*Bulk RNA‐Sequencing*: RNA extraction was completed by the Qiagen AllPrep DNA/RNA Mini kit (Qiagen, Hilden, Germany) according to manufacturer specifications. RNA‐sequencing was performed at the Johns Hopkins Experimental and Computational Genomics Core (ECGC). All RNA samples passed the quality control procedure using the Bioanalyzer Total RNA Pico kit (Agilent, Santa Clara CA). The libraries for RNA sequencing were constructed by TruSeq Stranded Total RNA with Ribo‐Zero (Illumina, San Diego CA), which was monitored using the Bioanalyzer High Sensitivity kit (Agilent, Santa Clara CA). The libraries were sequenced on an Illumina Novaseq 6000 (Illumina, San Diego CA) instrument using 150bp paired‐end dual indexed reads to a target depth of ≈100000 000 reads per sample with Q30 > 92%. Reads were aligned to the reference genome utilizing illumine package bcl2fastq. Differential expression analysis and statistical testing were performed using DESeq2 software. The biological functions were investigated via KEGG pathway analysis and Gene Set Enrichment Analysis (GSEA) comparing the expression of different genes (DGE).

### TCGA Analysis

Pancreatic cancer bulk RNA‐seq data were downloaded from the TCGA database, and the expression of genes were cleanout and normalized for subsequent analysis.

### In Vitro Biological Function Assay

*Mast Cell and Treg Migration Assay*: To perform the mast cell migration assay, we seeded the upper chamber of 3µm uncoated 12‐well Boyden chamber (BD Biosciences, USA) with 2 × 10^5^ mMCs in 400µL of completed DMEM. The lower chamber was filled with 750µL of completed DMEM as a control, as well as the indicated tumor‐conditioned medium. After 12 hours, the filters were removed and the number of migrated mast cells was counted. All migration assays were done in triplicate.

### CCK‐8, Colony Formation, EDU, and Apoptosis Assay

In the CCK‐8 assay, PDAC cell lines were seeded in 96‐well plates and cultured in 100µL of appropriate medium with or without 100ng mL^−1^ rhCXCL10. After that, 10µL CCK‐8 solution was added to each well, and the plates were incubated for one hour. The absorbance at 450nm was then measured at indicated time points after cell inoculation. The data represents the mean ± standard deviation (SD) from five independent experiments.

For the colony formation assay, the cells were seeded in 6‐well plates and cultured in medium with or without 100ng mL^−1^ rhCXCL10. Depending on the colony growth, the cells were cultured for one to two weeks. After that, the cells were fixed with 4% paraformaldehyde and stained with crystal violet to count the number of colonies.

In the EDU assay, the cells were seeded in six‐well plates and cultured in an appropriate medium with or without 100ng/mL rhCXCL10 for 48 h. The proliferated cells were then detected using EDU assay according to the manufacturer's instructions and visualized by immunofluorescence microscope (Olympus, Japan).

For the apoptosis assay, the cells were seeded in six‐well plates and cultured in an appropriate medium with or without 100ng mL^−1^ rhCXCL10 for 48 h. After that, the cells were digested into single cells and stained using an apoptosis kit following the manufacturer's instructions. The early and later apoptosis rate were analyzed by a flow cytometer Arial II (BD Biosciences, USA).

### Cell Stemness and Mobility Evaluation

After indicated treatment, tumor cells were resuspended in DMEM/F12 containing 20ng mL^−1^ rhEGF, 1×B27, and 100ng mL^−1^ bFGF, and 200 cells per well were sed into 96‐well ultra‐low attachment plate (Corning, USA) for 14 days culturing. The number of sphere clone that diameter is over 75µm were calculated in each well, and the sphere formulation efficacy was calculated by sphere number/200.

For migration assay, transwell assays were perform in an 8‐µm size. Tumor cells were seed in the uncoated upper chamber with 300µL FBS‐free medium, while 750µL of completed DEME medium was added to the lower chamber. After 24 h of incubation, the cells were fixed with 4% paraformaldehyde for 20 min and stained with crystal violet. The cells in the upper chamber were then removed. Invading cells were photographed using a microscope (Olympus, Japan) in three random views, and the average number of cells was calculated. The experiment was repeated three times for biological replication.

For invasion assay, 8µm transwell chamber were coated by matrix gel with pre‐cold pipette tips and kept in cell incubator for solidification. Then indicated tumor cell suspensions without FBS‐free medium were added to the upper chamber and completed medium was added into the lower chamber. After incubation for 48 hours, the chambers were harvested for invasion ability evaluation similarly to migration assay.

### Transfection and Luciferase Reporter Assay

CFPAC‐1 and PANC‐1 cells were seed in 6‐well plates 24 h before transfection. They were transfected with PPARγ promoter luciferase reporter and Renilla‐luciferase plasmid (as a control) using lipofectamine 3000. Similarly, mast cells were co‐transfected with wt‐CXCL10/mut‐CXCL10 promoter luciferase reporter plasmid and ERG overexpression plasmid. Two days after transfection, PDAC cells were stimulated by CXCL10 for 48 h and lysed for luciferase activity assay according to the manufacturer's instructions. Mast cells were also lysed for dual‐luciferase reporter assay.

### Mast Cell Proliferation and Cell Cycle Assay

To perform the mast cell proliferation assay, mast cells were pre‐stained with CFSE in a concentration of 2.5uM for 10min at 37 °C and washed by PBS for three times. Then, the cells were seeded in a 10cm dish, and cultured in DMEM or tumor‐conditioned medium supplemented with 10ng mL^−1^ SCF, 10ng mL^−1^ IL‐3, and 10% (v/v) FBS. The cells were cultured for 48 h and subsequently collected for analysis using the Arial II flow cytometer.

For the cell cycle assay, mast cells were seeded in a 10cm dish and cultured for 48 h in DMEM or tumor‐conditioned medium supplemented with 10ng mL^−1^ SCF, 10ng mL^−1^ IL‐3, and 10% (v/v) FBS. The cells were then collected and fixed with ethanol. After fixation, the cells were digested by RNase (100µg mL^−1^) and washed with PBS. Finally, the cells were stained by PI (50µg mL^−1^) for 30min and analyzed by the Arial II flow cytometer.

### Cell Death and Ferroptosis Analysis

Mast cells were cultured in tumor‐conditioned medium or DMEM as a control for 24 h. Afterward, they were stimulated with the indicated concentration of Erastin for 24 h. The cells were then collected and incubated with C11 BODIPY (1mg mL^−1^) for 20 min to analyze lipid ROS, or incubated with PI (5µg mL^−1^) / DAPI (10µg mL^−1^) for 5 min to analyzed cell death, or incubated with DCFH‐DA (10uM) for 30 min to analyze total ROS.

To evaluate ferroptosis, cells were cultured in tumor‐conditioned medium or DMEM as a control for 24 h. They were then stimulated with the indicated concentration of Erastin for 6 h. After collection, the cells were incubated with FerroOrange for 30 min, and analyzed using a flow cytometer or fluorescence microscope.

### Exosome Identification and Intake Assay

KPC‐derived exosomes were collected from 50 mL KPC conditioned medium and purified through ultra‐centrifugation. In brief, the medium was centrifuged at 2000g for 10 min and then at 10 000g for 10 min to remove cells and debris. The supernatant was further centrifuged at 100 000g for 90 min to precipitate and collect the exosomes for observation under a transmission electron microscope (Hitachi, Japan) and analysis through western blot.

The purified exosomes were labeled using PKH26 labeling kit. Briefly, the exosomes were mixed with PKH26, which was diluted in Reagent C, and washed to remove any unconjugated dye. The labeled exosomes were subsequently resuspended for the mast cells intake assay and observed using a fluorescence microscope.

### Pre‐Clinical Murine Model Experiments

*Establishment of Subcutaneous/Orthotopic Murine PDAC Model*: Female C57BL/6 mice, six weeks old, were purchased from JieSiJie Laboratory Animal Co, Ltd and kept in a specific pathogen‐free (SPF) atmosphere. To establish a subcutaneous tumor model, 100µL of KPC or Panc02 cell suspension (2 × 10^7^ mL^−1^) mixed with Matrigel was injected into the back subcutaneously. For the orthotopic tumor model, mice were anesthetized with 2% (m/v) pentobarbital sodium solution and 50µL KPC or Panc02 cell suspension (2 × 10^7^ mL^−1^) mixed with Matrigel was injected into the pancreas tail. The tumor volume was calculated by 1/2× (long diameter) × (short diameter)2.

### Mast Cells Adaptive Transfer Experiment

Mast cells were isolated from C57BL/6 mice and maintained in vitro until purified, as mentioned above. Lentivirus was used to construct CXCL10 knockdown mast cells, and transfection efficiency was determined by qRT‐PCR analysis. For the mast cell transplantation assay, sh‐CXCL10 knockdown (CXCL10^sh1/sh2^) or negative control (CXCL10^NC^) MCs (10^7^ mast cells per mouse) were injected into the tail vein of tumor burden mice at 3, 6, and 9 days after pancreatic cancer inoculation.

### Monotherapy or Combination Therapeutics

The indicated treatments were started three days after tumor inoculation. SCG was dissolved in PBS buffer and administrated intraperitoneally at dose of 75mg kg^−1^ three times a week. Gemcitabine (hydrochloride formulation) was dissolved in PBS buffer and administrated intraperitoneally at dose of 25mg kg^−1^ once a week. αPD‐1 antibody was dissolved in PBS buffer and administrated intraperitoneally at dose of 3mg kg^−1^ once a week.

### Optical Imaging for Tumor Burden Evaluation and Survival Analysis

Optical imaging was used to detect tumor cells transfected with luciferase using an in vivo imaging system (CleVue™ Vieworks, Korean). D‐luciferin sodium salt was intraperitoneally injected at a dose of 150mg kg^−1^ per mouse, and luciferase activity was evaluated within 30min after injection.

For survival analysis, PDAC orthotopically inoculated mice received the indicated treatment and were monitored until they succumbed spontaneously or were humanely terminated via euthanasia upon the appearance of severe physical symptoms such as severe cachexia, ascites, hypothermia, and tumor size exceeding 2 cm.

### Histological Pathology

At the end of animal experiment, the mice were sacrificed humanistically. Tumor tissues were harvested and fixed in 4% paraformaldehyde. Tissues were routinely embedded in paraffin and sliced into sections for H&E or immunohistochemistry staining. The detailed manufacturer's information of the reagents above is listed in Table S6 (Supporting Information).

### Statistical Analysis

All statistical analyses were performed by SPSS 22.0 and visualized by GraphPad Prism 9 software (GraphPad, San Diego, CA, USA). After Kolmogorov‐Smirnov normality test, two‐tailed Student's t‐test or one‐way ANOVA analysis was used for statistics. Post hoc LSD or Tamhane method were further used in subgroup analysis for one‐way ANOVA analysis. Non‐parametric test was applied for non‐normally distribution. All data were presented as mean with standard deviation. Kaplan‐Meier curves with log‐rank tests were used to analyze survival, and the univariate and multivariate Cox proportional hazards regression models were applied to analyze the independent prognostic indicators for OS and RFS. All the in vitro assays were repeated at least three times with biological replicates. The number of independent biological replicates was represented by n value in the figure legends or dots in the figures. Statistical significance was considered as P values <0.05.

### Ethics Approval

This study involves samples from human participants approved by the Ethics Committee of Zhongshan hospital, Fudan University (B2023‐129). Participants gave informed consent to participate in the study prior to enrolment and the consent obtained directly from patients. All animal experiments were approved by Animal Ethics Committee of Zhongshan Hospital, Fudan University (Y2022‐702).

## Conflict of Interest

The authors declare no conflict of interest.

## Author Contributions

H.Y., Q.C., and S.G. contributed equally as co‐first authors in this article. H.Y., Q.C., S.G., and N.P. performed conceptualization, data curation, and wrote the draft, formal analysis, and methodology. S.S., Y.X., J.R.H., T.H., W.G., J.W., L.Z., H.X., C.S., J.H., W.W., Y.J., and M.G.G. performed formal analysis and methodology. N.P., S.G., W.W., and L.L. performed funding acquisition. N.P., J.Y., W.W., W.L., and L.L. performed supervision and reviewed the draft. All authors have read and approved the article.

## Supporting information



Supporting Information

## Data Availability

The data that support the findings of this study are available from the corresponding author upon reasonable request.
