# Supplementary material for: The Crosstalk with CXCL10‐Rich Tumor‐Associated Mast Cells Fuels Pancreatic Cancer Progression and Immune Escape
Source: Adv Sci (Weinh). 2025 Feb 18;12(14):2417724. doi: 10.1002/advs.202417724 (PMC11984875; doi:10.1002/advs.202417724)
Supplement: Supplementary file 1 — Supporting Information [file ADVS-12-2417724-s001.docx]

**Supporting Information**

**
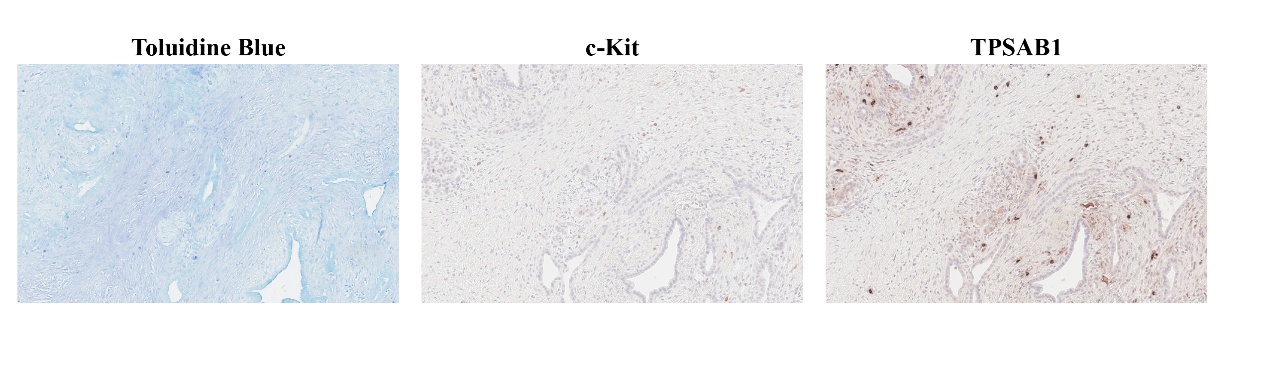
**

**Figure S1.** Optimum marker for tumor-associated mast cell staining. Representative images of serial slide of PDAC tissues with toluidine blue staining, c-kit or TPSAB1 immunohistochemistry staining.


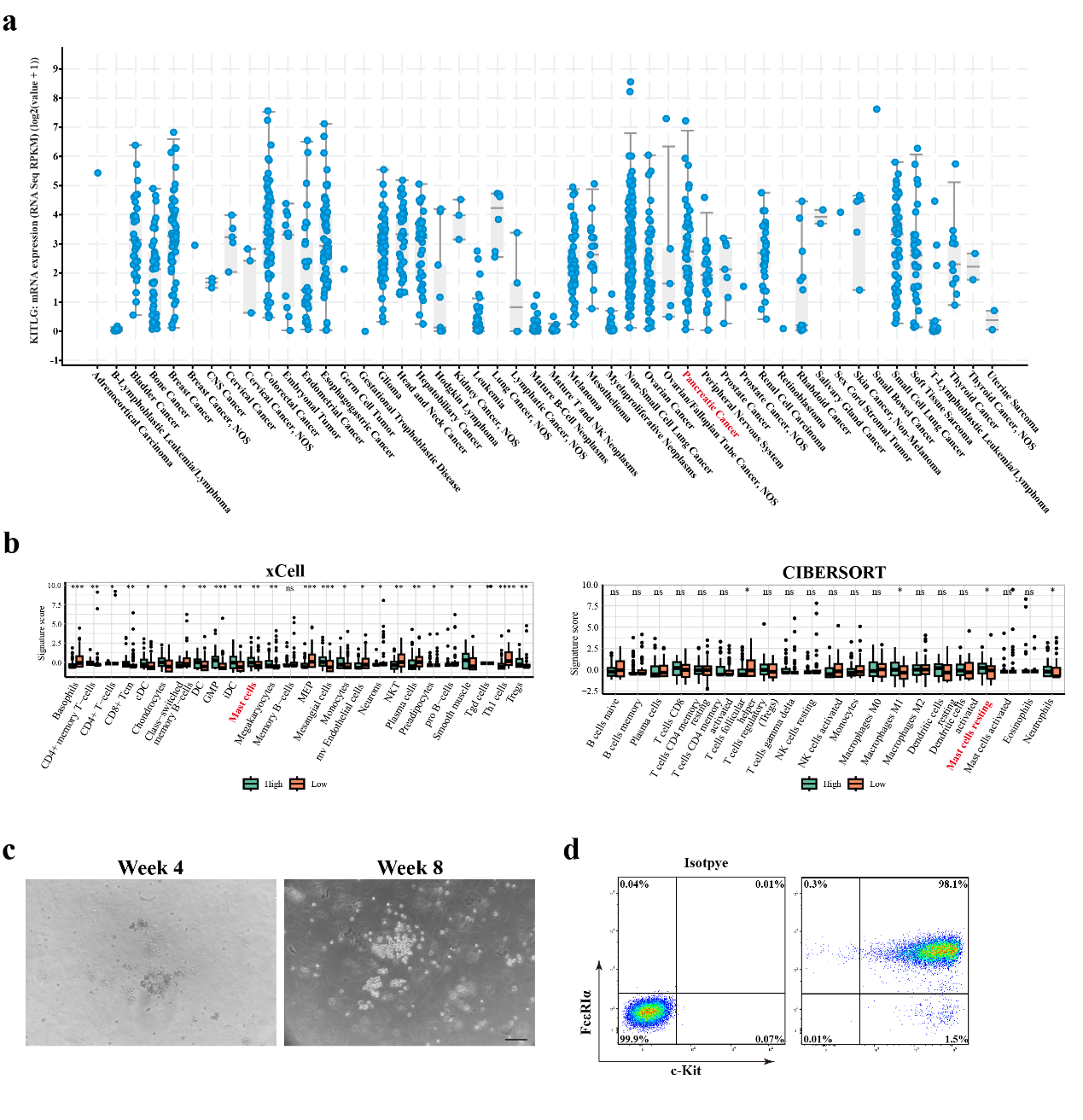


**Figure S2.** SCF is constitutively expressed in PDAC cells and correlated with mast cell infiltrations. a) Analysis of SCF expression within pan-cancer cell lines in the CCLE database. b) The relationship between *cxcl10* mRNA and tumor-infiltrating immune cells evaluated by xCell and CIBERSORT algorithm. c) Representative images of amplified hMCs *in vitro* at week 4 and week 8. Scale bar: 200μm. d) Flow cytometry analysis for identification of amplified hMCs at week 8. n.s. *P*>0.05, **P*<0.05, ***P*<0.01, and ****P*<0.001, Data were displayed as mean ± SD.


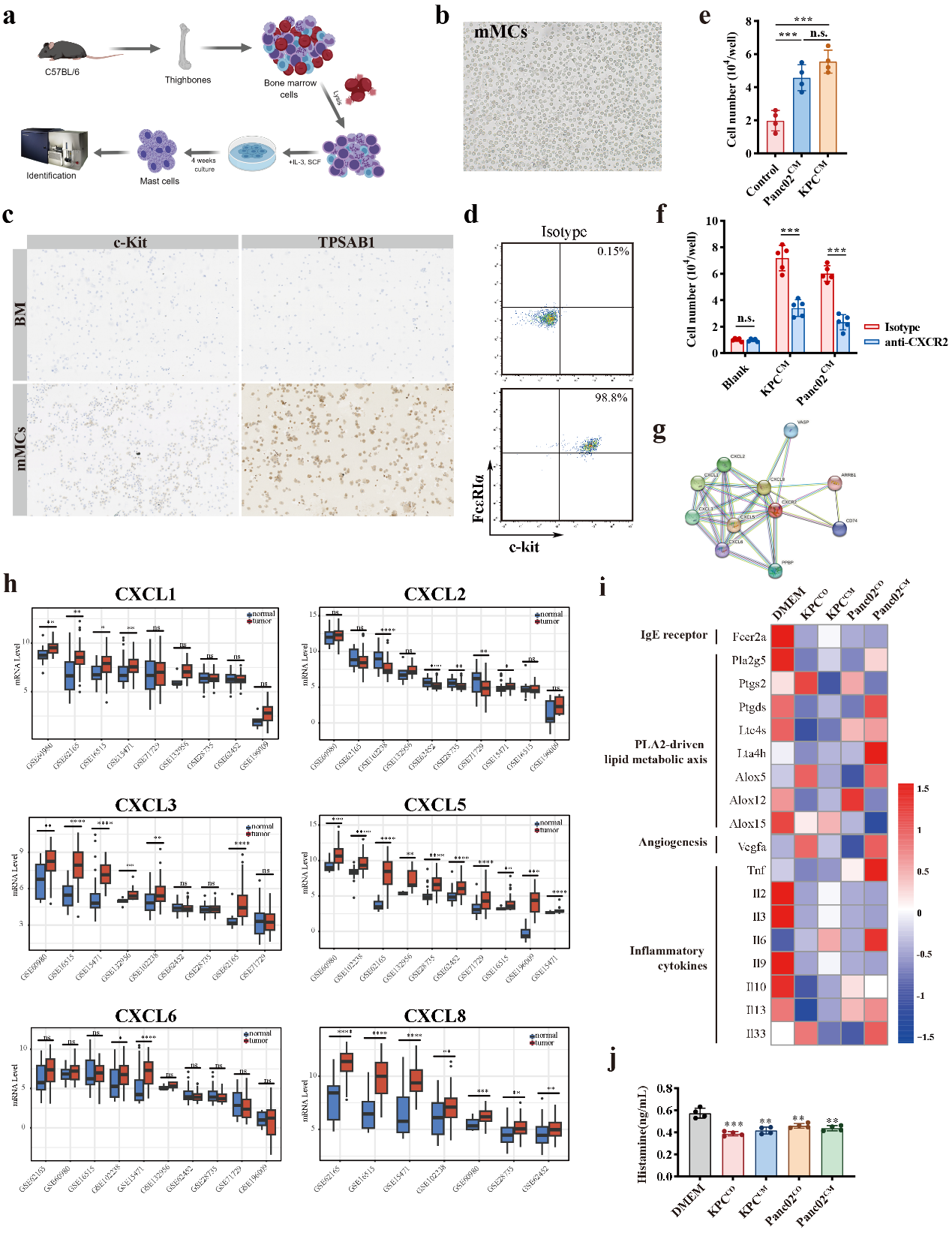


**Figure S3.** CXCR2^+^ mast cells are accumulated in PDAC. a) Schematic representation of mMC separation and amplification *in vitro*. b) Representative image of amplified mMCs at week 4. c) Identification images with c-kit and TPSAB1 immunohistochemistry staining for bone marrow (BM) and mMCs. d) Flow cytometry analysis for identification of amplified mMCs at week 4. e) The counts of mMCs migrated into the lower chamber under the indicated conditions. 2×10^5^ mMCs were seeded in the upper chamber in a transwell assay, and tumor-conditioned medium or DMEM as control were added into the lower chamber. Then, the cells in the lower chamber were harvested and calculated after 12 hours. n=4 per group. f) 10μg/mL CXCR2 antibody were used to treat mMCs or IgG isotype as control, and then chemotactic assay was performed similarly as above. g) The protein-protein interaction (PPI) network of CXCR2 was built and displayed by STRING (https://cn.string-db.org/). h) Analysis of differential expression of CXCR2 ligand between PDAC and adjacent normal tissues in ten public datasets. i) Heatmap of allergic-related gene expression in tumor-associated mast cells. j) Histamine was detected in tumor-associated mast cell supernatants. mMCs were treated by co-culturing with tumor cells or tumor-conditioned medium for 24 hours, then balanced by Tyrode buffer and stimulated by C48/80 (5μg/mL) for degranulation. n.s. *P*>0.05, **P*<0.05, ***P*<0.01, and ****P*<0.001, Data were displayed as mean ± SD.


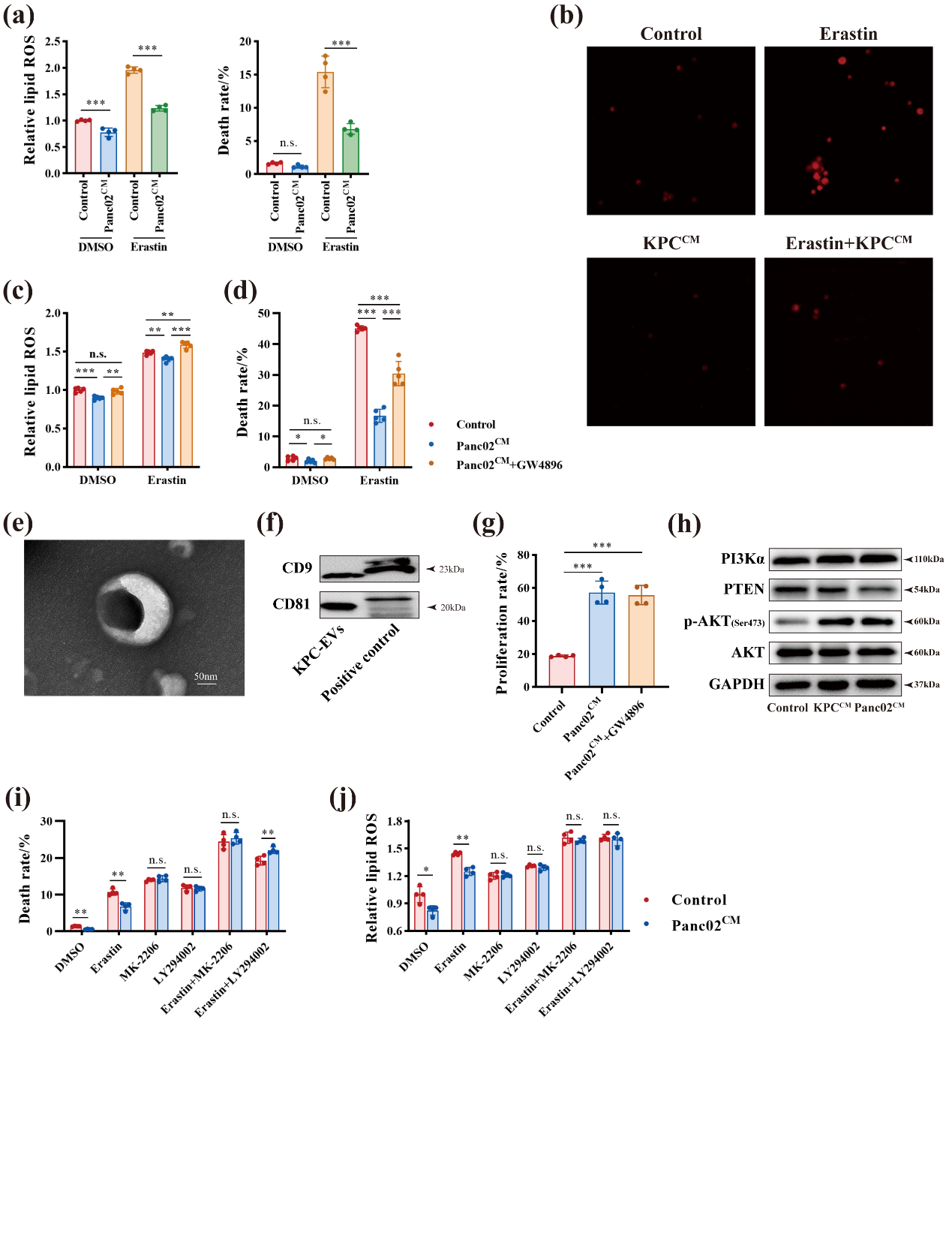


**Figure S4.** Tumor-derived exosomes activate AKT signaling and suppress ferroptosis of mMCs. a) Flow cytometry analysis for the ferroptosis in mMCs treated with Panc02-conditioned medium. mMCs were pretreated by conditioned medium for 12 hours and Erastin was added for additional 24 hours. Then, lipid ROS (left) and cell death rate (right) of mMCs were analyzed by C11-BODIPY and PI staining, respectively. n=4 per group. b) Representative images of FerroOrange staining in mMCs with different treatments. c) and d) Flow cytometry analysis for the lipid ROS levels (c) and cell death rates (d) in tumor-associated mast cells with or without exosomes. n=5 per group. e) The representative image of KPC-derived exosome via electron microscope. f) Western blot analysis for exosome markers from KPC supernatant. g) Flow cytometry analysis for the proliferation rate of mast cells by CFSE label after indicated treatment for 48 hours. mMCs were prelabeled by CFSE (1μM) for 10 minutes and stimulated by Panc02-conditioned medium with or without exosomes for 48 hours. n=4 per group. h) Western blot analysis for the AKT pathway activation in mMCs after 24 hours stimulation by tumor-conditioned medium. i) and j) Flow cytometry analysis for the lipid ROS levels (i) and cell death rates (j) in mMCs with indicated treatments. mMCs were pretreated by AKT inhibitors for 2 hours and followed by Panc02-conditioned medium treatment for 12 hours, then Erastin was added into indicated groups or DMSO as control for 24 hours. Student’s t test was used for comparison. n=4 per group. n.s. *P*>0.05, **P*<0.05, ***P*<0.01, and ****P*<0.001, Data were displayed as mean ± SD.


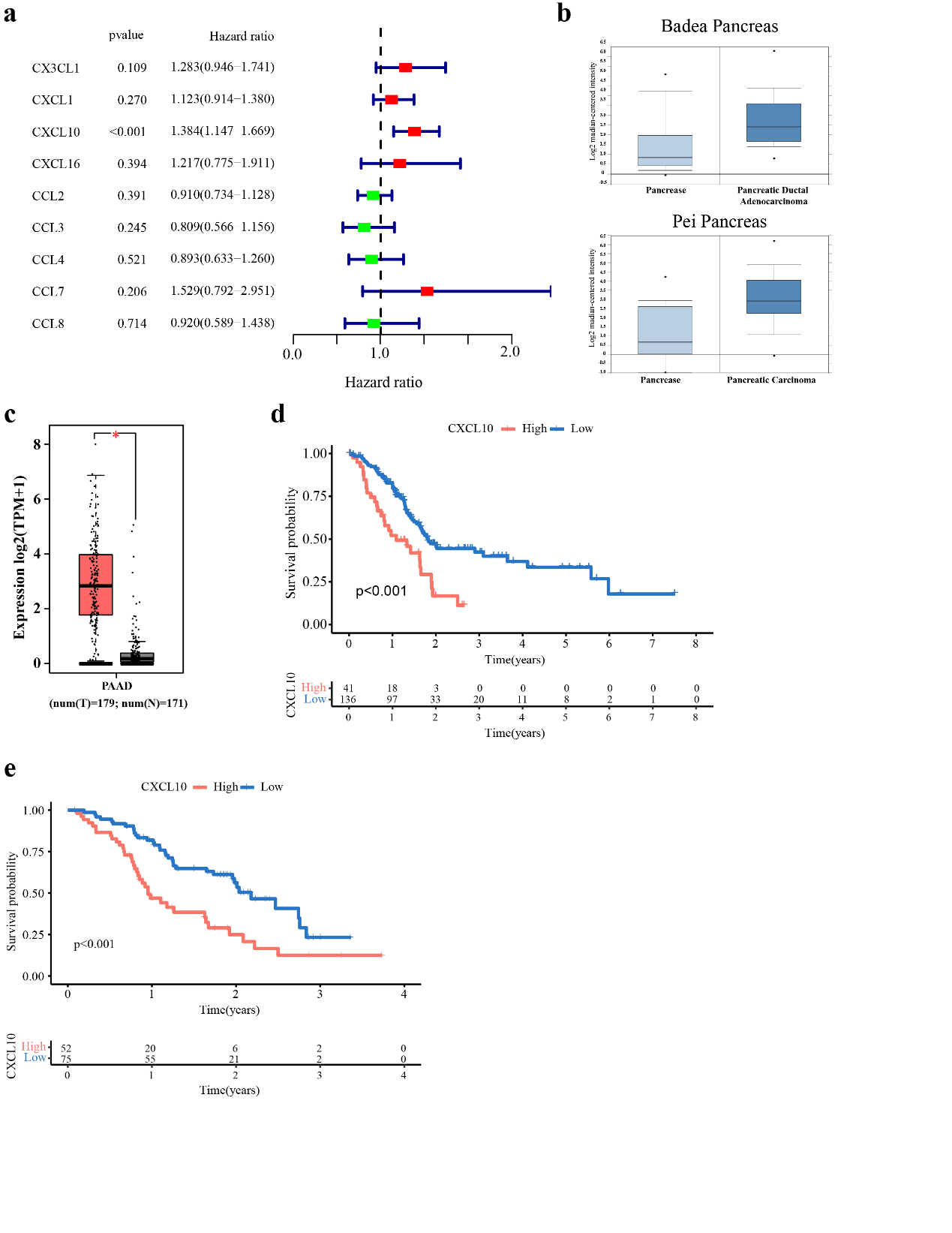


**Figure S5.** CXCL10 is associated with a poor prognosis in PDAC patients. a) Univariate analysis of prognostic value of chemokines for overall survival in PDAC patients in the TCGA database. b) Comparative analysis of *cxcl10* mRNA expression in PDAC and normal pancreas in the Oncomine database. c) Comparative analysis of *cxcl10* mRNA expression in PDAC and normal pancreas in the TCGA database by GEPIA (http://gepia.cancer-pku.cn/). d) and e) Kaplan-Meier analysis of *cxcl10* mRNA expression or CXCL10 protein level in PDAC for overall survival in the TCGA (d) and in CTPAC databases (e) separately. Survival curves were analyzed by log-rank tests.


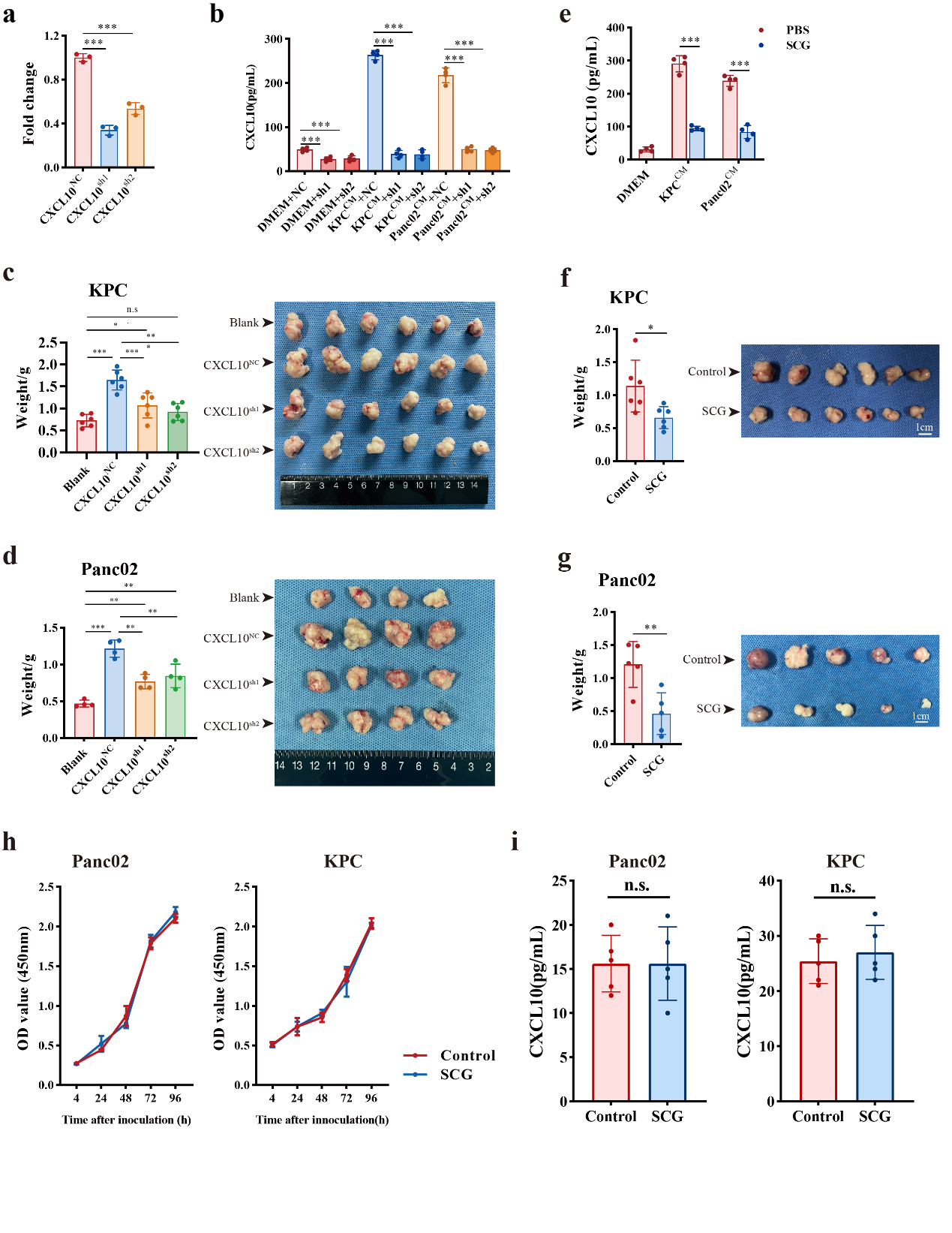


**Figure S6.** Tumor-associated mast cell-derived CXCL10 promotes tumor progression, which was inhibited by SCG *in vivo*. a) and b) qRT-PCR (a, n =3) and ELISA (b, n=4) analysis for CXCL10 levels after *cxcl10* knockdown in mMCs by short hairpin RNA. c) and d) Analysis of tumor weights (left) and images of tumor masses (right) in KPC (c, n=6) and Panc02 (d, n=4) orthotopic tumor models receiving engineered mMC adoptive transfer or not. e) ELISA analysis for CXCL10 in tumor-associated mast cell supernatant with or without 10μg/mL SCG pretreatment for 24 hours. f) and g) Analysis of tumor weights (left) and images of tumor masses (right) in KPC (f, n=6) and Panc02 (g, n=5) subcutaneous tumor models receiving SCG treatment in C57BL/6. h) The CCK-8 assay analysis for the effect of 10μg/mL SCG on tumor growth *in vitro*. i) CXCL10 concentration in tumor supernatant with or without 10μg/mL SCG treatment *in vitro*. n.s. *P*>0.05, **P*<0.05, ***P*<0.01, and ****P*<0.001, Data were displayed as mean ± SD.


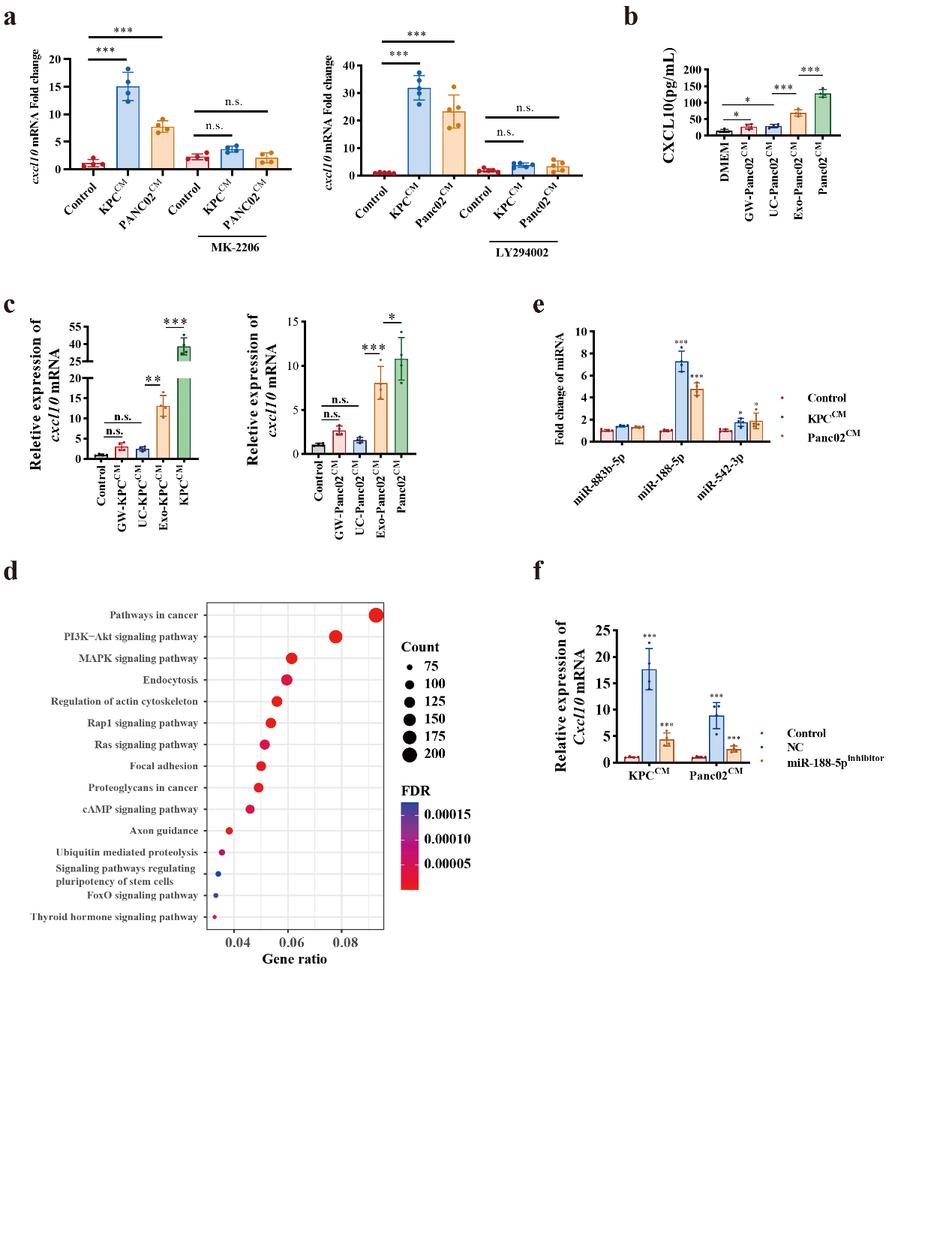


**Figure S7.** Tumor-derived exosome miR-188-5p promotes AKT pathway activation and *cxcl10* expression in mast cells. a) qRT-PCR analysis for *cxcl10* mRNA expression in tumor-associated mMCs with or without MK-2206 (left, n=4) and LY294002 (right, n=5) pretreatment, DMSO were used as control. b) ELISA assay analysis for supernatant CXCL10 level from tumor-associated mast cells stimulated by Panc02-conditioned medium with or without exosomes. n=4 per group. c) qRT-PCR analysis for c*xcl10* mRNA expression of tumor-associated mMCs with or without exosomes stimulated by KPC or Panc02 conditioned medium. n=4 per group. d) Functional enrichment analysis of the target gene of KPC-derived exosomes packaged miRNA via GSEA from public data (GSE95741). e) qRT-PCR analysis of the expression of three potential exosome-packaged miRNAs in mMCs after tumor conditioned medium for 24 hours. n=4 per group. f) qRT-PCR analysis for the expression of *cxcl10* mRNA in mMCs with negative control transfected tumor conditioned medium, miR-188-5p inhibited tumor conditioned medium or DMEM as control. n=4 per group. n.s. *P*>0.05, **P*<0.05, ***P*<0.01, and ****P*<0.001, Data were displayed as mean ± SD.


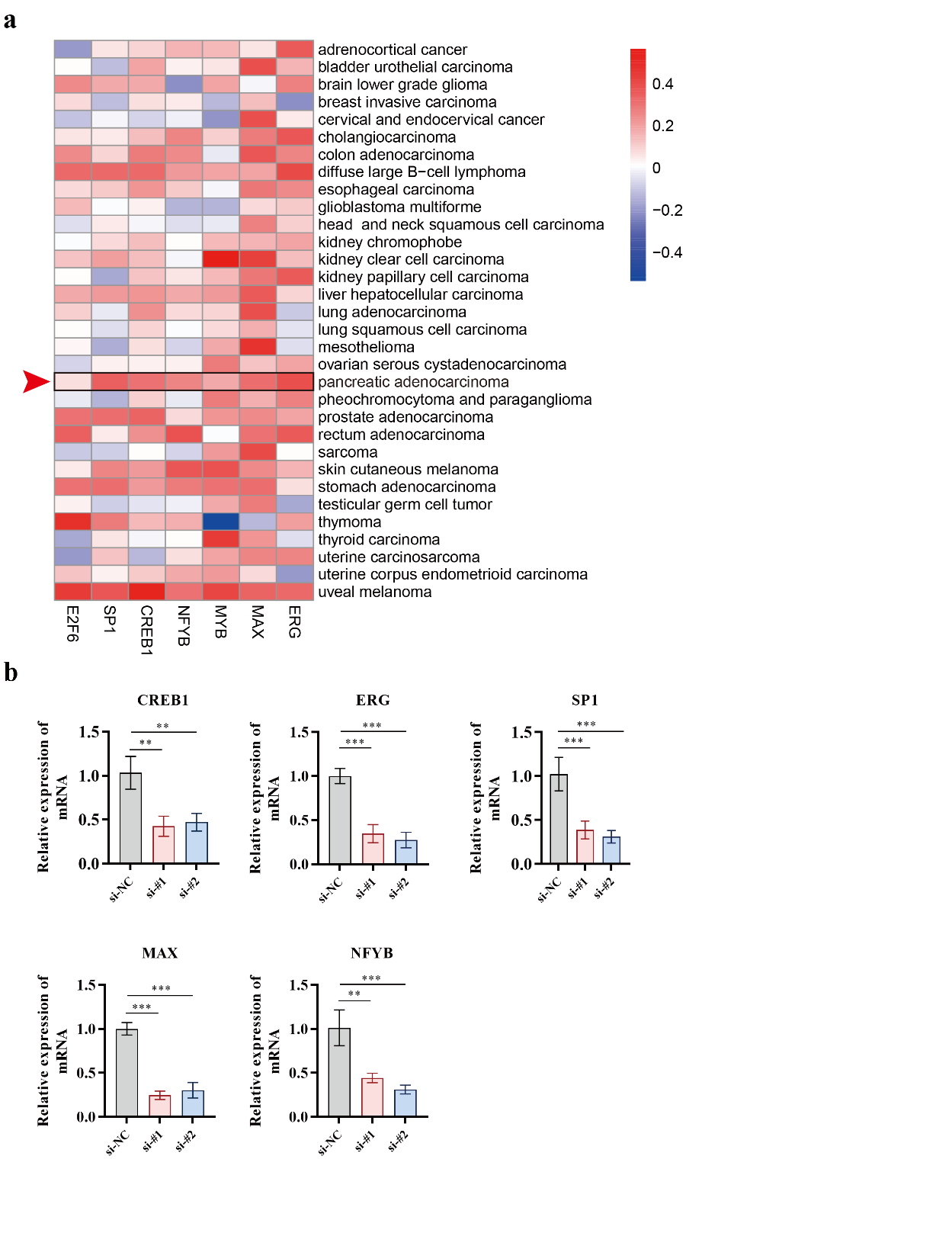


**Figure S8.** Candidate transcriptional factors for *cxcl10* mRNA transcription in tumor-associated mast cells. a) Heatmap showing the correlations between the *cxcl10* mRNA and levels of seven transcriptional factors in pan-cancer from the TCGA database. b) qRT-PCR analysis for the relative mRNA expression of the potential transcriptional factors after siRNA transfection in mMCs. Student’s t test was used for comparison. n=3 per group. ***P*<0.01, and ****P*<0.001, Data were displayed as mean ± SD.


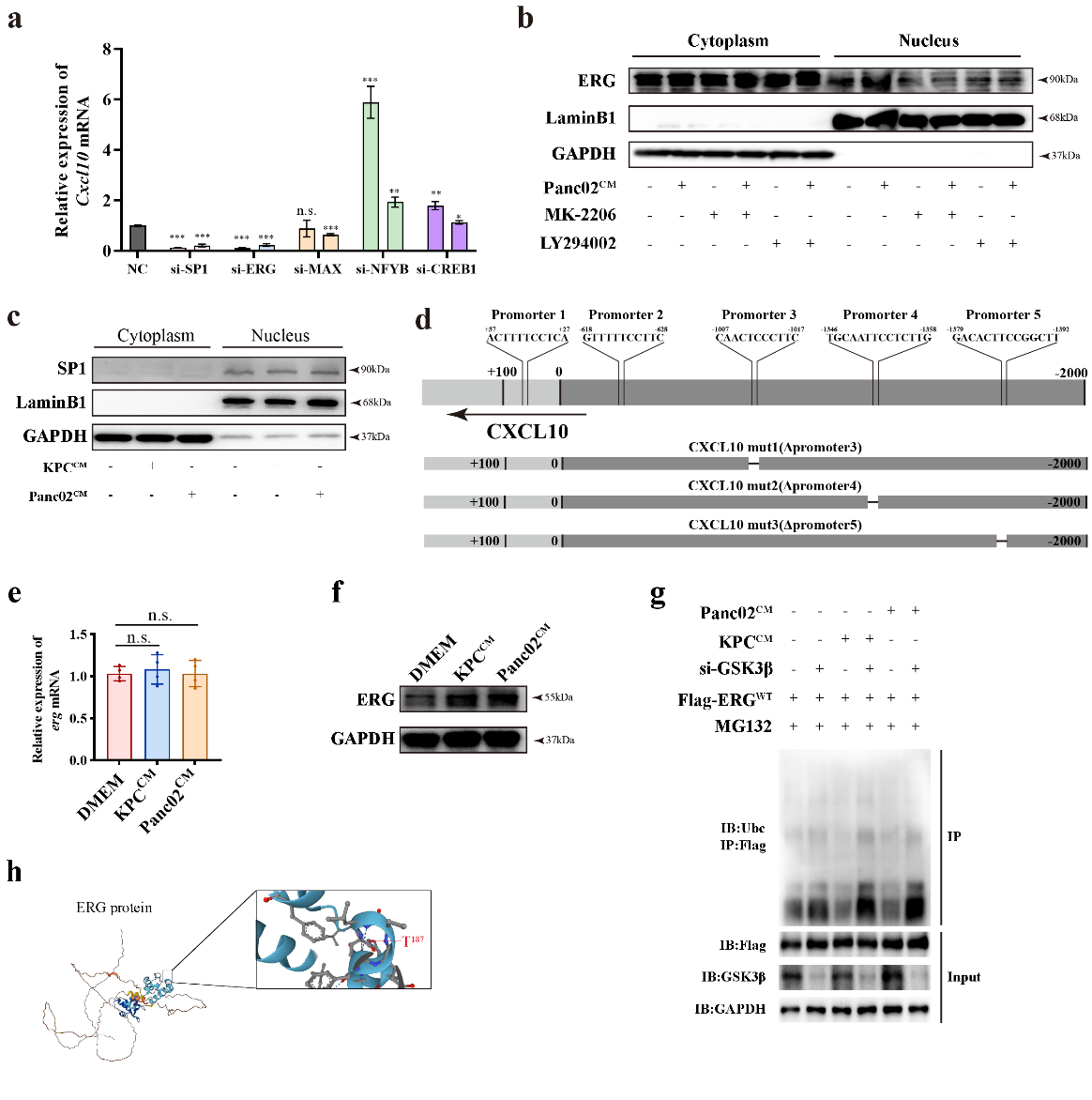


**Figure S9. ERG is the core transcriptional factor that mediates *cxcl10* mRNA transcription.** a) qRT-PCR analysis for the relative mRNA expression after knockdown of the potential transcriptional factors in mMCs. n=3 per group. b) Western blot analysis for ERG nuclear translocation in tumor-associated mast cells treated by AKT inhibitors. c) Western blot analysis for SP1 nuclear translocation in tumor-associated mast cells. d) The schematic images of potential binding sites of ERG to CXCL10 promoter. e) and f) qRT-PCR (e) and western blot (f) analysis for ERG expression in tumor-associated mMCs. n=4 per group. g) Western blot analysis for the ubiquitination level of ERG protein in tumor-associated mMCs with or without GSK3β knockdown in the presence of MG132. h) The 3D structure of murine ERG analyzed by Alphafold (https://alphafold.ebi.ac.uk/). n.s. *P*>0.05, **P*<0.05, ***P*<0.01, and ****P*<0.001, Data were displayed as mean ± SD.


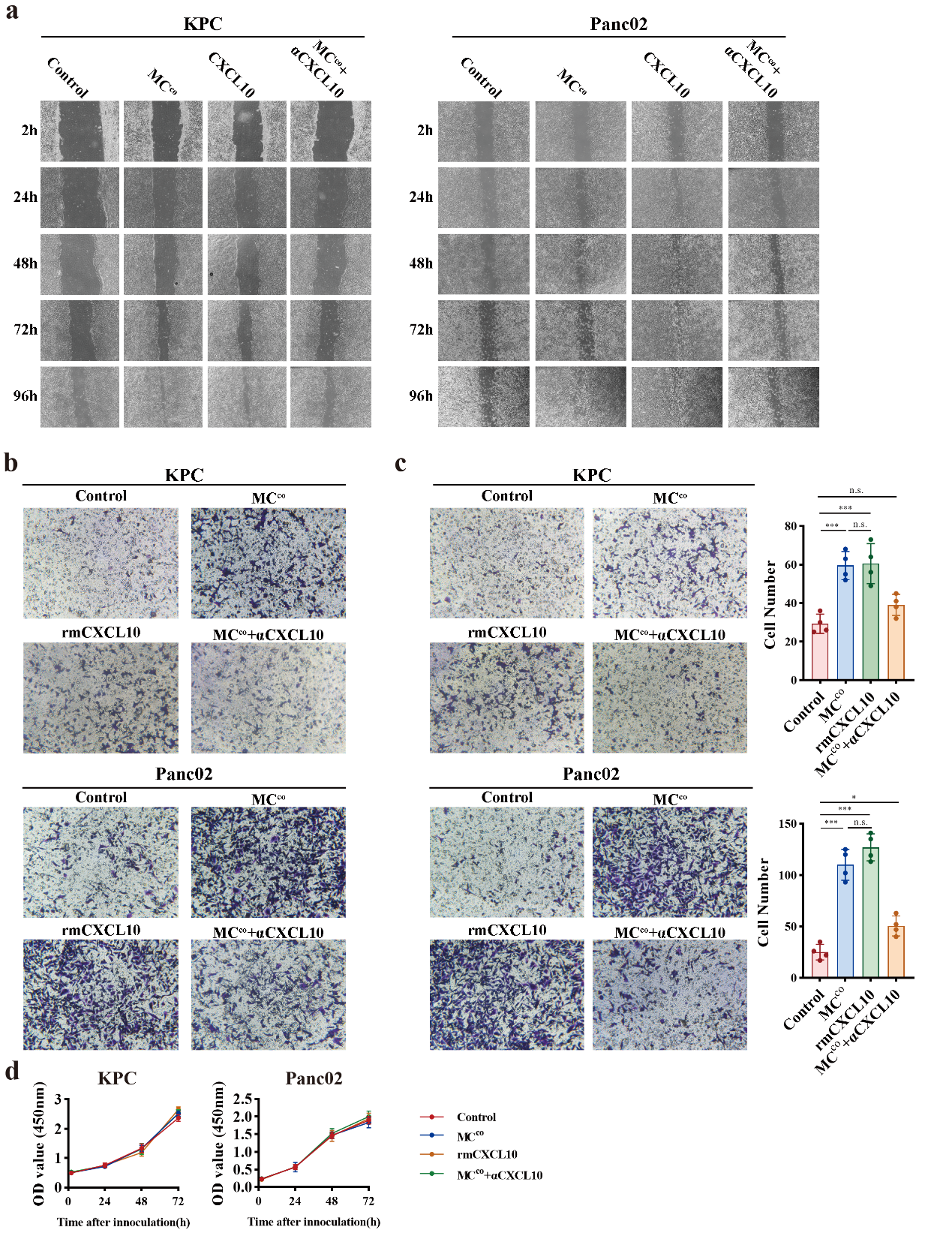


**Figure S10.** CXCL10 increases tumor mobility but not proliferation *in vitro*. a) and b) The representative images of wounding scratch assay (a) and transwell assay (b) in KPC and Panc02 cells after co-culturing with mMCs with or without CXCL10 blockade, and rmCXCL10 (100ng/mL) stimulation. c) The representative images of invasion assay in KPC and Panc02 cells after co-culturing with mMCs with or without CXCL10 blockade, and rmCXCL10 (100ng/mL) stimulation and statistical analysis. n=4 per group. d) CCK-8 assay analysis for growth rate of KPC and Panc02 cells *in vitro* after co-culturing with mMCs with or without CXCL10 blockade, and rmCXCL10 (100ng/mL) stimulation. n.s. *P*>0.05, **P*<0.05, ***P*<0.01, and ****P*<0.001, Data were displayed as mean ± SD.


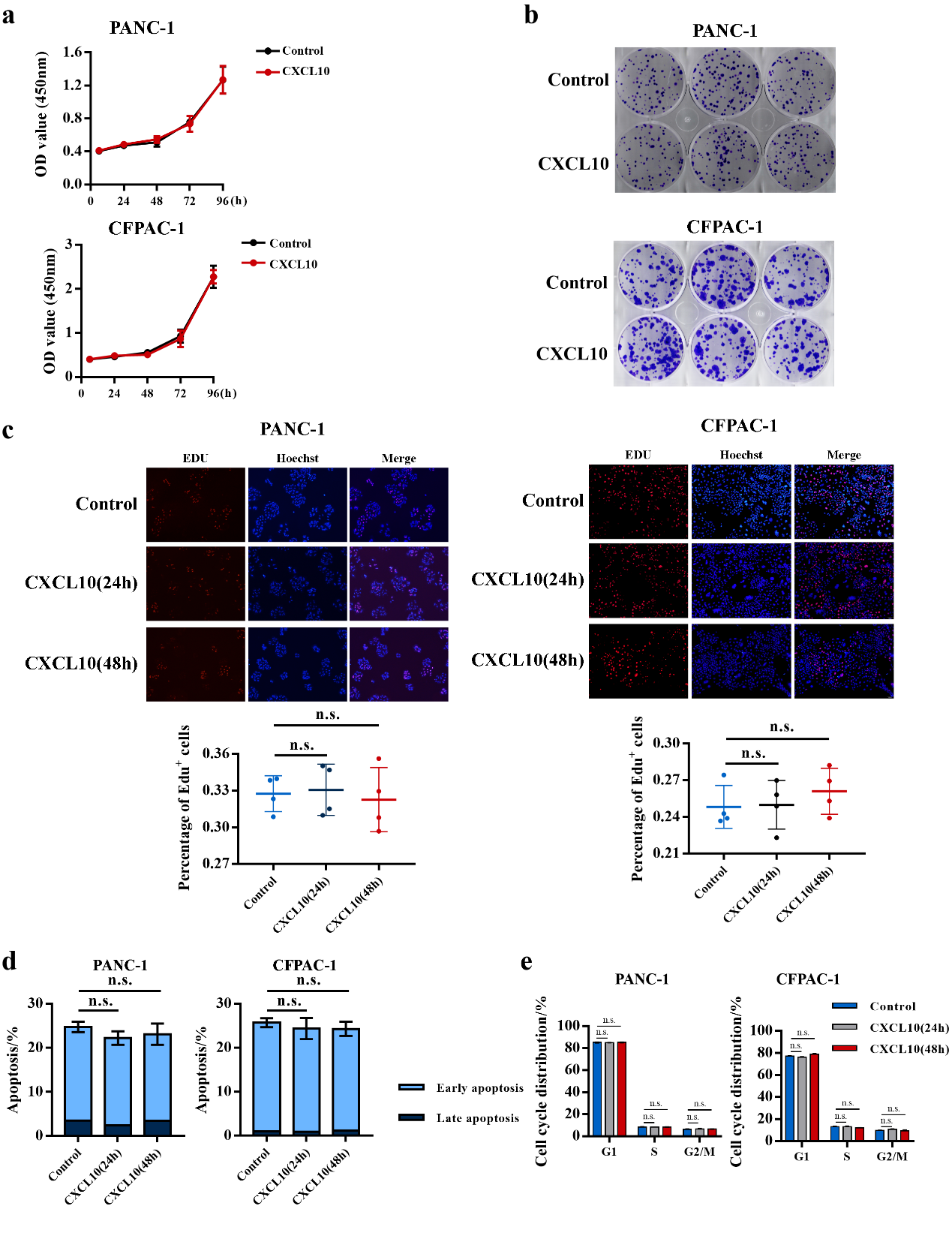


**Figure S11.** CXCL10 has no direct effect on tumor growth, apoptosis and cell cycle. a) - c) CCK-8 assay (a), colony assay (b) and EDU assay (c) analysis for tumor proliferation *in vitro* with rhCXCL10 (100ng/mL) stimulation in PANC-1 and CFPAC-1 cells. d) Flow cytometry analysis for the apoptotic rates with rhCXCL10 (100ng/mL) stimulation in PANC-1 and CFPAC-1 cells. n=4 per group. e) Flow cytometry analysis for cell cycle with rhCXCL10 (100ng/mL) stimulation for 48 hours in PANC-1 and CFPAC-1 cells. n=4 per group. n.s. *P*>0.05, **P*<0.05, ***P*<0.01, and ****P*<0.001, Data were displayed as mean ± SD.


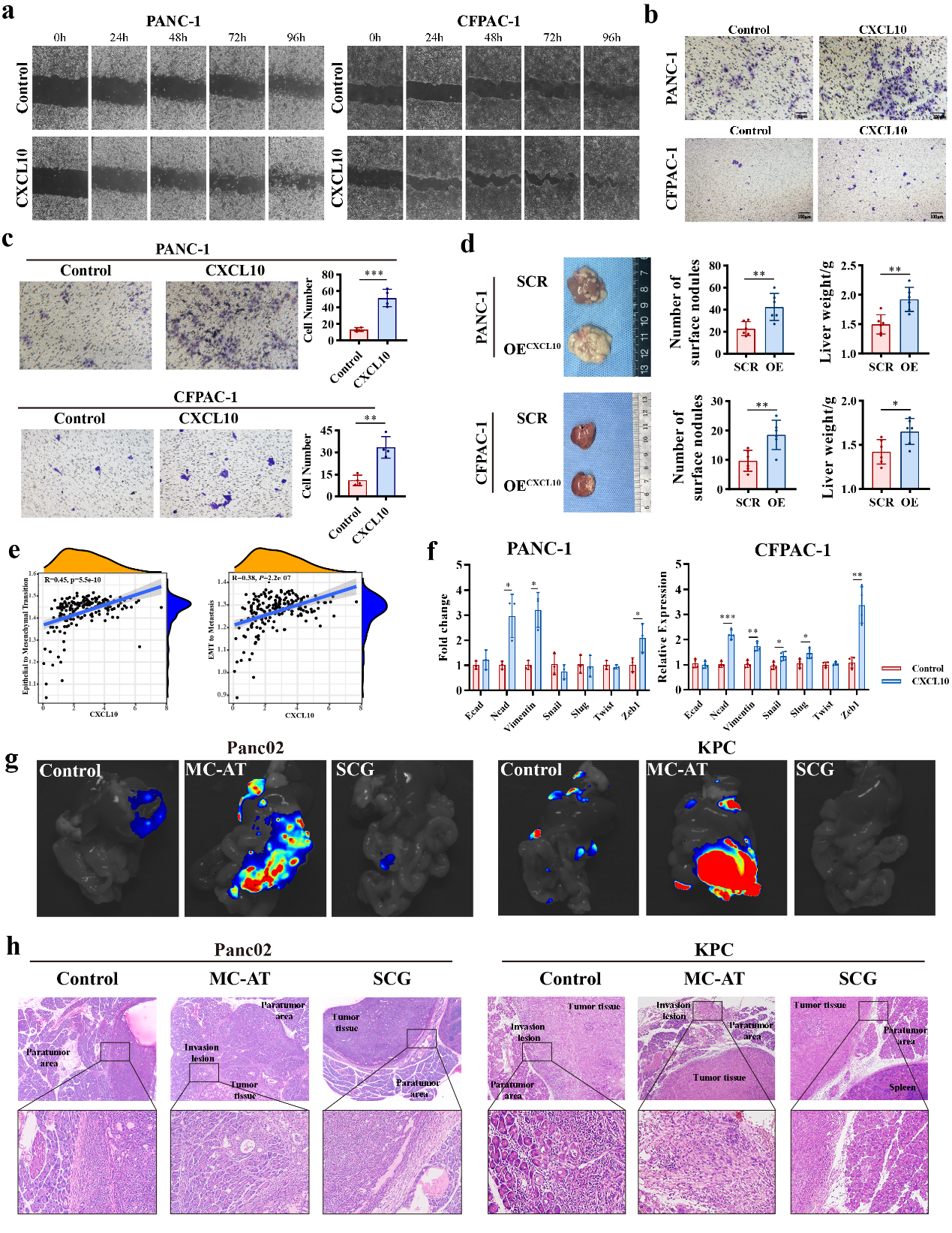


**Figure S12.** Tumor-associated mast cell-derived CXCL10 promotes tumor invasion and migration, which is inhibited by SCG. a) and b) Representative images of wounding scratch assay (a) and migration assay (b) to evaluate PANC-1 and CFPAC-1 mobility *in vitro* after rhCXCL10 (100ng/mL) stimulation. c) The representative image and statistical analysis of invasion assay in PANC-1 (left) and CFPAC-1 (right) cells. d) The representative image of PDAC liver metastasis that established by splenic injection. In brief, PANC-1 or CFPAC-1 were constitutively overexpressed rhCXCL10 by lentivirus. Then, 2×10^6^ cells/50μL PBS engineered PANC-1 or CFPAC-1 were injected into mouse spleen. 40 days after inoculation, the liver was harvested for further analysis. e) The relationship between EMT signature scores and *cxcl10* expression in PDAC from the TCGA database (p <0.05, Spearman test). f) qRT-PCR analysis for the expression of EMT genes in PANC-1 and CFPAC-1 after rhCXCL10 stimulation for 24 hours. n=3 per group. g) Representative image of local recurrence and/or distant metastasis. Tumor cells were stably transfected with luciferase and established orthotopic tumor models. After measuring whole mice luciferase activity, the organ was dissected immediately to detect luciferase activity by IVIS imaging system to reduce the additional influences. The luciferase signals represent distant metastatic lesion or local recurrence. h) Representative HE staining images of resected tumor tissues with MC adoptive transfer or SCG treatment. n.s. *P*>0.05, **P*<0.05, ***P*<0.01, and ****P*<0.001, Data were displayed as mean ± SD.


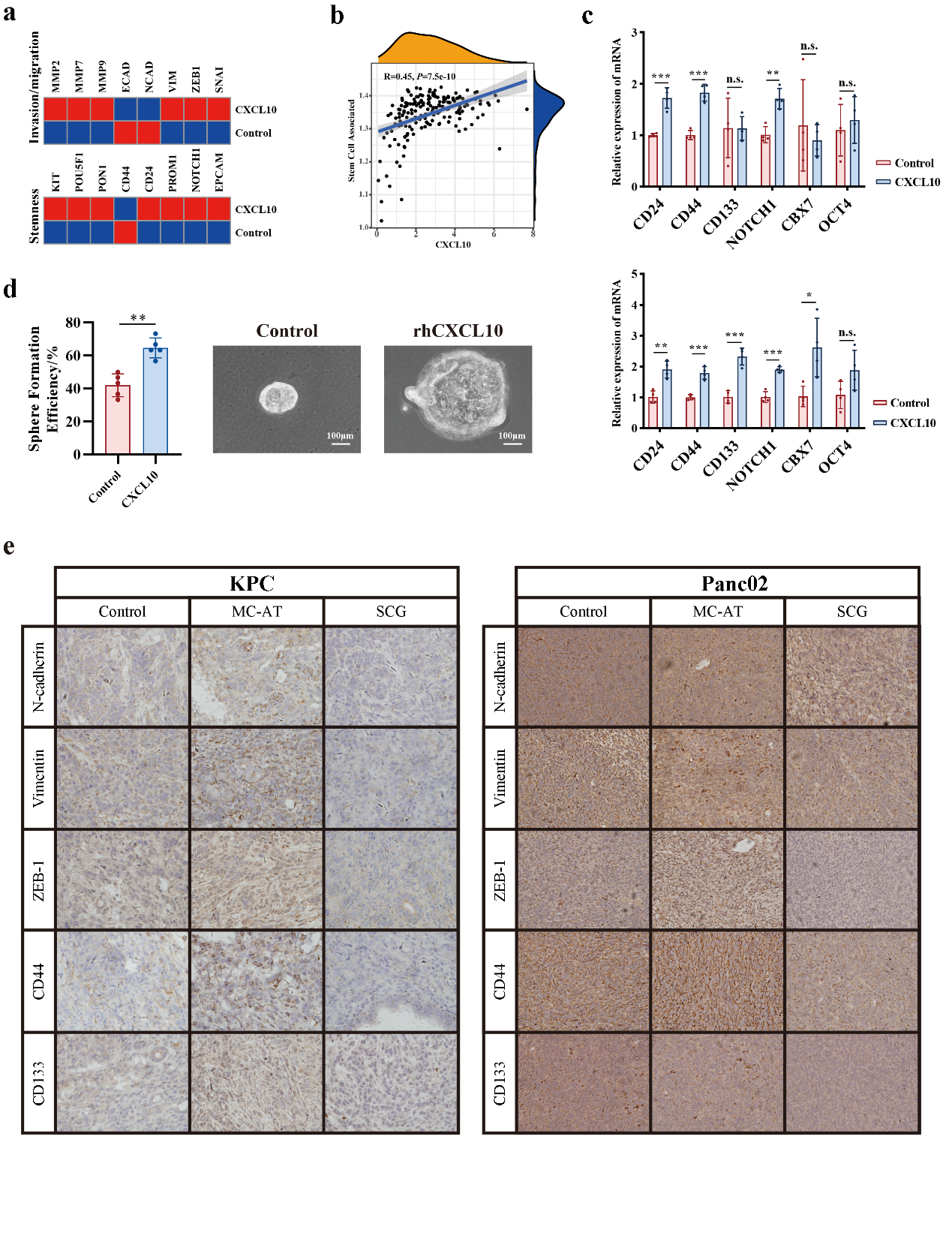


**Figure S13.** CXCL10 promotes the EMT and stemness of PDAC. a) Heatmap of the mobility and stemness-related gene expressions in CXCL10-treated cells and controls. b) The relationship between stemness signature score and *cxcl10* mRNA expression in PDAC from the TCGA database (*P*<0.05, Spearman test). c) qRT-PCR analysis for the expression of stemness genes after rhCXCL10 stimulation for 24 hours in PANC-1 and CFPAC-1. n=4 per group. d) Sphere formation assay (left) and representative images of CFPAC-1 cell cluster (right) after pretreating with rhCXCL10 or not for 24 hours. Student’s t test was used for comparison. n=4 per group. e) Immunohistochemistry staining images for resected tumor tissues with MC adoptive transfer, SCG treatment or not. n.s. *P*>0.05, **P*<0.05, ***P*<0.01, and ****P*<0.001, Data were displayed as mean ± SD.


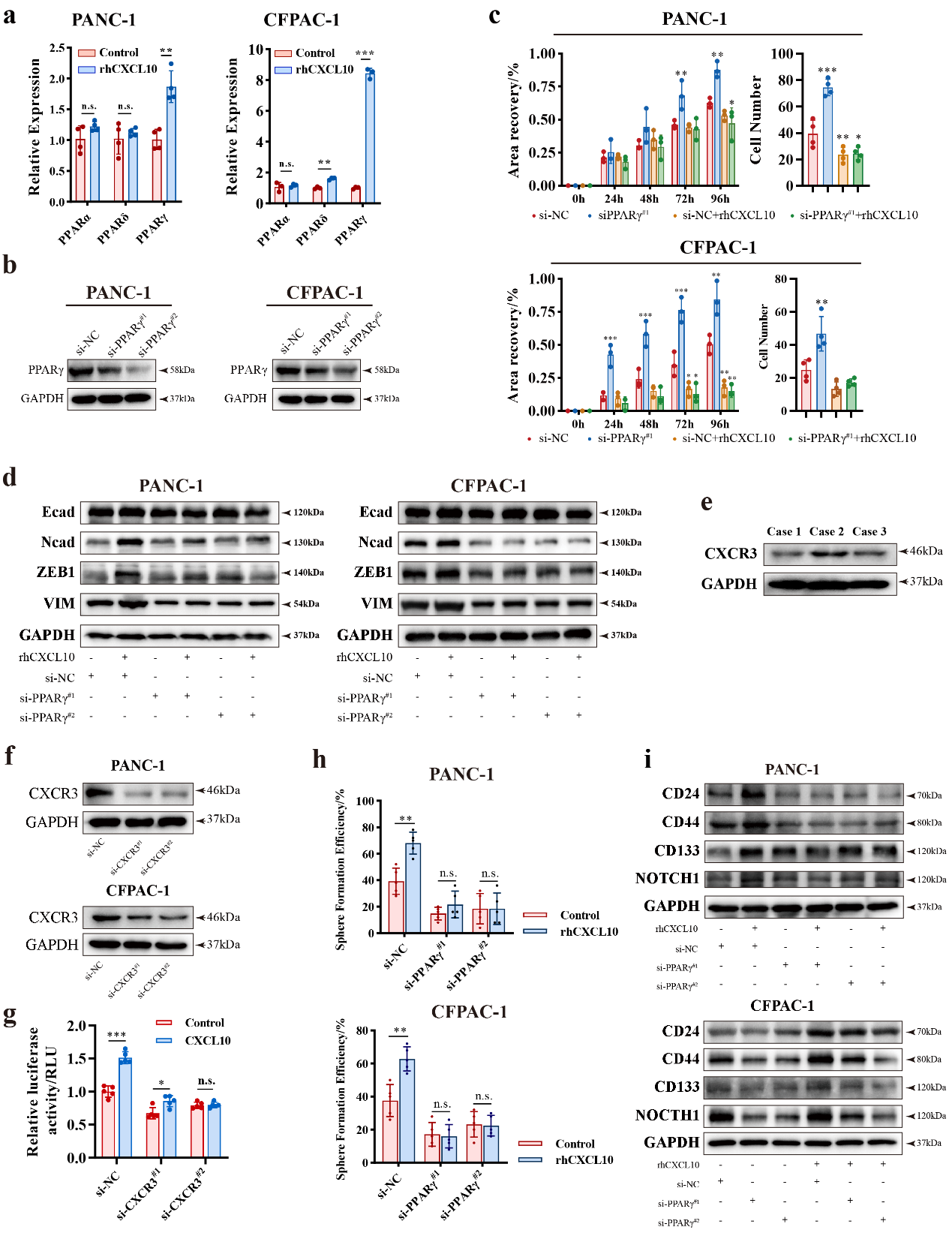


**Figure S14.** CXCL10 promotes PDAC migration and stemness via CXCR3-PPARγ axis. a) qRT-PCR analysis for the expression of PPAR isoforms after rhCXCL10 stimulation after 24 hours in PANC-1(n=4) and CFPAC-1(n=3). b) Western blot analysis for identification of PPARγ knockdown in PANC-1 and CFPAC-1 after PPARγ siRNA transfection. c) Quantification of migration by scratch assay (left panel) and transwell assay (right panel) in PPARγ knockdown PANC-1 and CFPAC-1 with rhCXCL10 stimulation or not. d) Western blot analysis for the expression of EMT-related genes in PANC-1 and CFPAC-1 after PPARγ knockdown and rhCXCL10 stimulation. e) Western blot analysis for identification of CXCR3 expression on three PDAC patients derived organoids. f) Western blot analysis for CXCR3 expression in PANC-1 and CFPAC-1 after CXCR3 siRNA transfection. g) Dual-luciferase assay analysis for rhCXCL10 effect on PPAR transcriptional activity in PANC-1 and CFPAC-1 after CXCR3 knockdown. n=5 per group. h) Sphere formation assay analysis for the effect of rhCXCL10 on the stemness of PANC-1 and CFPAC-1 after PPARγ knockdown. n=5 per group. i) Western blot analysis for stemness-related gene expressions in PANC-1 and CFPAC-1 after PPARγ knockdown and rhCXCL10 stimulation. n.s. *P*>0.05, **P*<0.05, ***P*<0.01, and ****P*<0.001, Data were displayed as mean ± SD.


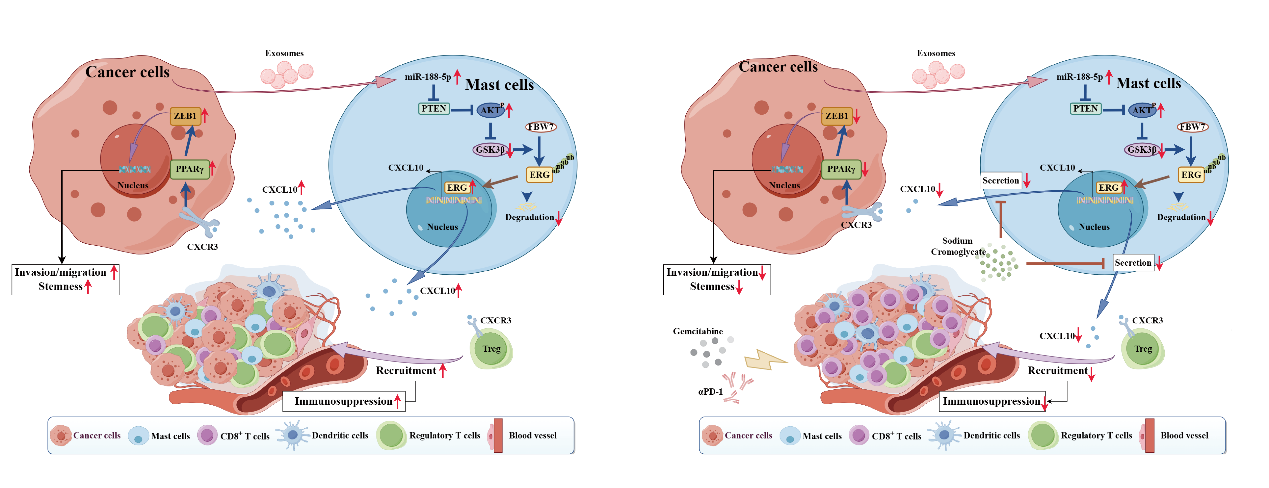


**Figure S15.** Representative schematic of the crosstalk between PDAC cells and tumor-associated mast cells.

**Table S1.** The relationships between tumor-infiltrating mast cell density and clinicopathological characteristics.

| **Characteristics** | **Low density** | **High density** | ***P* value** |
| --- | --- | --- | --- |
|  | **(n=54)** | **(n=36)** |  |
| **Gender** |  |  | 0.859 |
| Male/ Female | 34/20 | 22/14 |  |
| **Age (years)** |  |  | 0.255 |
| <70/ ≥70 | 36/18 | 28/8 |  |
| **Primary site** |  |  | 0.163 |
| Head/ Body or tail | 28/26 | 24/12 |  |
| **Differentiation** |  |  | **0.020** |
| I/ II/ III | 1/22/31 | 0/6/30 |  |
| **Microvascular invasion**  No/Yes | 49/5 | 35/1 | 0.438 |
| **Nerve invasion**  No/Yes | 7/47 | 3/33 | 0.732 |
| **T classification** |  |  | 0.770 |
| ≤4cm/ > 4cm | 39/15 | 27/9 |  |
| **N classification** |  |  | 0.930 |
| N0/N1-2 | 32/22 | 21/15 |  |
| **TNM stage**  I/II/III | 24/21/9 | 17/13/6 | 0.960 |
| **Fibrinogen** |  |  | 0.564 |
| ≤400/ > 400 mg/dl | 44/10 | 31/5 |  |
| **CA19-9** |  |  | 0.548 |
| < 37/≥37 U/L | 12/42 | 10/26 |  |
| **CEA** |  |  | 0.689 |
| < 5/ ≥ 5 ng/mL | 40/14 | 28/8 |  |
| **TBIL** |  |  | 0.534 |
| ≤ 20.4/>20.4 μmol/L | 35/19 | 21/15 |  |
| **Albumin** |  |  | 0.822 |
| < 35/ ≥ 35 g/L | 10/44 | 6/30 |  |
| **ALT** |  |  | 1.000 |
| ≤ 35/ > 35 U/L | 30/24 | 20/16 |  |
| **AST** |  |  | 0.861 |
| ≤ 40/ > 40 U/L | 32/22 | 22/14 |  |
| **GGT** |  |  | 0.085 |
| ≤ 60/ > 60 U/L | 23/21 | 22/14 |  |
| **ALP** |  |  | 0.792 |
| ≤ 125/ > 125 U/L | 33/21 | 21/15 |  |
| **LDH** |  |  | 0.812 |
| ≤245/ >245 U/L | 46/8 | 30/6 |  |
| **Glucose** |  |  | 0.067 |
| ≤ 5.6/ >5.6 mmol/L | 27/27 | 11/25 |  |

**Table S2.** The univariate and multivariate analysis of prognostic factors associated with overall survival and recurrence-free survival.

| **Characteristics** | **Patients** | **Overall survival** | | | **Recurrence-free survival** | | |
| --- | --- | --- | --- | --- | --- | --- | --- |
|  |  | **Univariate *P* value** | **Multivariate *P* value** | **HR (95% CI)** | **Univariate *P* value** | **Multivariate *P* value** | **HR (95% CI)** |
| **Total** | 90 |  |  |  |  |  |  |
| **Gender** |  |  |  |  |  |  |  |
| Male/Female | 56/34 | 0.650 | NA |  | 0.512 |  |  |
| **Age (years)** |  |  |  |  |  |  |  |
| <70/≥70 | 64/26 | **0.015** | 0.097 | 0.579 (0.304-1.104) | **0.010** | 0.091 | 0.597 (0.329-1.086) |
| **Primary site** |  |  |  |  |  |  |  |
| Head/Body or tail | 52/38 | 0.755 | NA |  | 0.647 |  |  |
| **T classification** |  |  |  |  |  |  |  |
| ≤4cm/>4cm | 66/24 | 0.183 | NA |  | 0.181 |  |  |
| **N classification** |  |  |  |  |  |  |  |
| N0/N1-2 | 53/37 | **<0.001** | **0.009** | 2.751 (1.284-5.896) | **<0.001** | 0.096 | 1.789 (0.902-3.548) |
| **TNM stage** |  |  |  |  |  |  |  |
| I/II/III | 41/34/15 | **<0.001** | 0.495 | 1.189 (0.723-1.956) | **<0.001** | 0.232 | 1.321 (0.837-2.087) |
| **Fibrinogen** |  |  |  |  |  |  |  |
| ≤400/>400 mg/dl | 75/15 | 0.463 | NA |  | 0.122 |  |  |
| **CA19-9** |  |  |  |  |  |  |  |
| < 37/ ≥ 37 U/L | 22/68 | 0.447 | NA |  | 0.823 |  |  |
| **CEA** |  |  |  |  |  |  |  |
| < 5/ ≥ 5 ng/mL | 68/22 | 0.499 | NA |  | 0.396 |  |  |
| **TBIL** |  |  |  |  |  |  |  |
| ≤ 20.4/ > 20.4 μmol/L | 56/34 | 0.805 | NA |  | 0.866 |  |  |

**Table S3.** The target sequences of siRNAs used in the study.

| Target | sense strand | antisense strand |
| --- | --- | --- |
| Murine siERG-1 | CCAUCAAGAUGGAGUGCAA | UUGCACUCCAUCUUGAUGG |
| Murine siERG-2 | CGAUGUGGACGUCUUACUA | UAGUAAGACGUCCACAUCG |
| Murine siSP1-1 | GCAGAAAGAGGGAGAGCAA | UUGCUCUCCCUCUUUCUGC |
| Murine siSP1-2 | GGGAAGAGCCUCAGGAGAU | AUCUCCUGAGGCUCUUCCC |
| Murine siMAX-1 | CAUAGAAGCUCUUGGACAA | UUGUCCAAGAGCUUCUAUG |
| Murine siMAX-2 | GGCCCAAAUCCUAGACAAA | UUUGUCUAGGAUUUGGGCC |
| Murine siNFYB-1 | UAAAUUCACUCACACAUUCCU | GAAUGUGUGAGUGAAUUUAUA |
| Murine siNFYB-2 | AAGGUUAUUACUGAAAAGGGA | CCUUUUCAGUAAUAACCUUUC |
| Murine siGSK3β | AGAAAGUUCUACAGGACAAGC | UUGUCCUGUAGAACUUUCUUG |
| Human siCXCR3-1 | AGAAGUUGAUGUUGAAGAGGG | CUCUUCAACAUCAACUUCUAC |
| Human siCXCR3-2 | AGAUGAAGUUUUAGUUUCCAA | GGAAACUAAAACUUCAUCUUC |
| Human siPPARγ-1 | GAAGACAUUCCAUUCACAA | UUGUGAAUGGAAUGUCUUC |
| Human siPPARγ-2 | CAGAUUGAAGCUUAUCUAU | AUAGAUAAGCUUCAAUCUG |

**Table S4.** The sequences of the primers for qRT-PCR used in this study.

|  | Forward primer | Reverse primer |
| --- | --- | --- |
| Murine MAX | CCTGGGCCGTAGGAAATGAG | CAGCCGCAGATTGAAACCTC |
| Murine CREB1 | ATCTGGAGCAGACAACCAGC | TGAGCTGCTGGCATGGATAC |
| Murine ERG | TGGCGAAACCAAGGACAATCA | TGCGGTCATCTCTGTCTTAGC |
| Murine NFYB | TGTCCAACCAAACAGCCGAT | ACAGTTTCAGAGGCTCCACG |
| Murine SP1 | CAAGTGCTGCCGTCATTTTCT | AGGATAGGCTCGCAGGGATG |
| Murine CXCL10 | CAAGTGCTGCCGTCATTTTCT | AGGATAGGCTCGCAGGGATG |
| Murine GAPDH | TGGGTGTGAACCACGAGAAA | CCCTTCCACAATGCCAAAGT |
| Murine CCR2 | CCTCAGTTCATCCACGGCAT | AGGGAGTAGAGTGGAGGCAG |
| Murine CCR4 | TGCTGGGTGGAGGAAATCAC | TCTCTACGCTTGTAACCAGGC |
| Murine CCR5 | AGACATCCGTTCCCCCTACA | GCAGGGTGCTGACATACCAT |
| Murine CCR7 | TGGCTCTCCTTGTCATTTTCCA | TCCGTCATGGTCTTGAGCCT |
| Murine CXCR1 | CAGCTGGTGCCTCAGATCAAA | ATCTCCAGTGGGCAGCATTC |
| Murine CXCR2 | GTTGGGAGCCACTCTGCTCA | GTTCAGCAGGTAGACATCGGT |
| Murine CXCR4 | AAACCTCTGAGGCGTTTGGT | TGCCGACTATGCCAGTCAAG |
| Murine CXCR7 | ACCGTCAGGAAGGCAAACCA | ACCGTCAGGAAGGCAAACCA |
| Human SCF | TCATTCAAGAGCCCAGAACCC | ACTGCTACTGCTGTCATTCCT |
| Human GAPDH | CTGACTTCAACAGCGACACCC | TTGCTGTAGCCAAATTCGTTG |
| Human PPARα | CCTCGGTGACTTATCCTGTGG | CATTCGTCCAAAACGAATCGC |
| Human PPARδ | CATCGGGCTTCCACTACGG | CCAGCTTCATGCGGATCGTA |
| Human PPARγ | CAGAAATGCCTTGCAGTGGG | CTTTCCTGTCAAGATCGCCCT |
| Human Ecadherin | TCATGAGTGTCCCCCGGTAT | TCTTGAAGCGATTGCCCCAT |
| Human Ncadherin | GCCATCAAGCCTGTGGGAAT | GGAGCCACTGCCTTCATAGT |
| Human Vimentin | TCCGCACATTCGAGCAAAGA | TGAGGGCTCCTAGCGGTTTA |
| Human ZEB1 | GTACCAGAGGATGACCTGCCA | GCCCTTCCTTTCCTGTGTCAT |
| Human Snail | TAGCGAGTGGTTCTTCTGCG | AGGGCTGCTGGAAGGTAAAC |
| Human Slug | ATCACTGTGTGGACTACCGC | TCACTCGCCCCAAAGATGAG |
| Human Twist | TTCAAAGAAACAGGGCGTGG | CAGAGGTGTGAGGATGGTGC |
| Human CD24 | GCTCCTACCCACGCAGATTT | GAGACCACGAAGAGACTGGC |
| Human CD44 | TGGACAGGACAGGACCTCTT | AGGTCCTGCTTTCCTTCGTG |
| Human CD133 | TCCACAGATGCTCCTAAGGC | CGCGGCTGTACCACATAGAG |
| Human NOTCH1 | TGAATGGCGGGAAGTGTGAA | ACTTGTACTCCGTCAGCGTG |
| Human CBX7 | TGCGGAAGGGTAAAGTCGAG | AAGCAGAGCTTCTCCTTGCC |
| Human OCT4 | CTTCGGATTTCGCCTTCTCG | CCTTGGAAGCTTAGCCAGGT |

**Table S5.** The sequences of the primers for ChIP-PCR used in this study.

| Primer for indicated promoter | Forward primer | Reverse primer |
| --- | --- | --- |
| Murine ERG-Promoter1 | ACTGGAATTACTCTTACGGCTT | TTGCACTGAATTATAGCAGATTAT |
| Murine ERG-Promoter2 | CTCAGTAAGGGACTATATATC | TGTAGGAACCTCAGAATTTTAG |
| Murine ERG-Promoter3 | CTGTCCCGTGATTTGCTC | CTGTCCCGTGATTTGCTC |
| Murine ERG-Promoter4 | GCGAGATCTATGTAACCTCACC | TAATTGTTGCCCCGTGCT |
| Murine ERG-Promoter5 | CTGTCCCGTGATTTGCTC | CTGTACCCACCAACGGGAA |

**Table S6.** The primary antibodies, recombinant proteins and reagents used in this study.

| Reagents | | |
| --- | --- | --- |
| Name | Company | Item Number |
| RPMI-1640 | Gibco | 11875093 |
| IMDM | Gibco | 12440053 |
| METHOCULT SF^BIT^ H4236 | Stem Cell Technologies Inc. | 04236 |
| Bovine serum albumin | Sigma-Aldrich | A-1595 |
| Insulin–transferrin–selenium supplement | Gibco | 51300-044 |
| 2-Mercaptoethanol | Gibco | 21985-023 |
| CD34 Progenitor Cell Isolation Kit | Miltenyi Biotec | 130-046-702 |
| DMEM | Gibco | 11965092 |
| FBS | Gibco | 10091148 |
| Toluidine Blue O | Sigma-Aldrich | T3260 |
| matrigel | Corning | 356231 |
| TrypLE™ Express Enzyme | Gibco | 12605010 |
| Cultrex Organoid Harvesting Solution | R&D stystems | [3700-100-01](https://www.rndsystems.com/cn/products/cultrex-organoid-harvesting-solution_3700-100-01) |
| Lipofectamine™ 3000 Transfection Reagent | Invitrogen | L3000075 |
| Cytoplasmic and nuclear seperation kit | Beyotime | P0028 |
| Protein A/G Magnetic Beads | MedChemExpress | HY-K0202 |
| DAPI | Beyotime | C1002 |
| PHK26 | Sigma-Aldrich | PKH26GL |
| Disodium Cromoglycate | Selleck | S1911 |
| Mouse CXCL10/IP-10/CRG-2 DuoSet ELISA | R&D stystems | DY466 |
| mmu-miR-188-5p Standard RNA | Ribobio | miRB0000217-2-1 |
| mmu-miR-883b-5p Standard RNA | Ribobio | miRB0004850-2-1 |
| mmu-miR-542-3p Standard RNA | Ribobio | miRB0003172-2-1 |
| mmu-miR-188-5p mimic | Ribobio | miR10000217-1-5 |
| micrON mimic NC | Ribobio | miR1N0000001-1-5 |
| mmu-miR-188-5p inhibitor | Ribobio | miR20000217-1-5 |
| micrOFF inhibitor NC | Ribobio | miR2N0000001-1-5 |
| LY294002 | Selleck | S1105 |
| MK-2206 | Selleck | S1078 |
| Compound 48/80 | Sigma-Aldrich | C2313 |
| B27 | Gibco | 17504044 |
| CD8^+^ T Cell Isolation Kit | Miltenyi Biotec | 130-096-495 |
| CellTrace™ CFSE Cell Proliferation Kit | Thermo Fisher | C34554 |
| PI | Beyotime | ST511 |
| C11-BODIPY | Thermo Fisher | D3861 |
| DCFA-DA | Sigma-Aldrich | 35845 |
| FerroOrange | DOJINDO LABORATORIES | F374 |
| FITC Annexin V Apoptosis Detection Kit | BD Biosciences | 556547 |
| Click-iT EdU cell proliferation assays | Thermo Fisher | C10339 |
| Dual Glo Luciferase Reporter Gene Assay Kit | Yeasen Biotechnology | 11405ES60 |
| D-Luciferin,Sodium Salt | Yeasen Biotechnology | 40901ES02 |
| SimpleChIP® Enzymatic Chromatin IP Kit | Cell Signaling Technology | 9003 |
| Gemcitabine hydrochloride | MedChemExpress | HY-B0003 |
